# Cyclometalated (N,C) Au(III) Complexes: The Impact of Trans Effects on Their Synthesis, Structure, and Reactivity

**DOI:** 10.1021/acs.accounts.3c00595

**Published:** 2023-12-05

**Authors:** Marte Sofie Martinsen Holmsen, Ainara Nova, Mats Tilset

**Affiliations:** † Department of Chemistry, University of Oslo, P.O. Box 1033, Blindern, N-0315 Oslo, Norway; ‡ Centre for Materials Science and Nanotechnology, University of Oslo, P.O. Box 1126, Blindern, N-0316 Oslo, Norway; ∥ Hylleraas Centre for Quantum Molecular Sciences, Department of Chemistry, University of Oslo, P.O. Box 1033, Blindern, N-0315 Oslo, Norway; ⊥ UiT-The Arctic University of Norway, N-9037 Tromsø, Norway

## Abstract

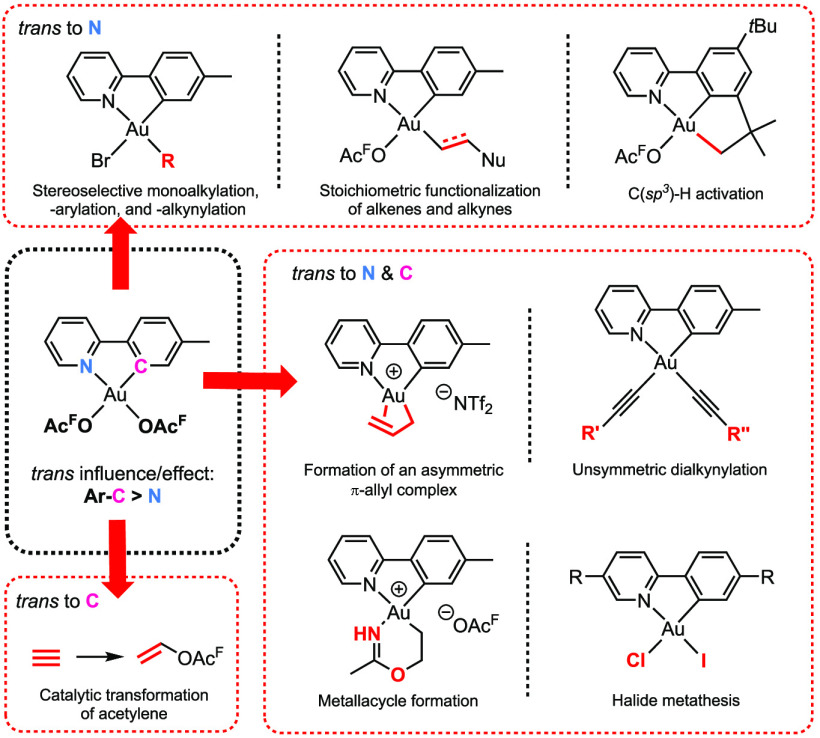

The early years of gold catalysis were dominated by Au(I) complexes and inorganic Au(III) salts. Thanks to the development of chelating ligands, more sophisticated Au(III) complexes can now be easily prepared and handled. The choice of the ancillary ligand has great consequences for the synthesis, properties, and reactivity of the Au(III) complex in question. Among the major factors controlling reactivity are the “trans effect” and the “trans influence” that a ligand imparts at the ligand trans to itself. The kinetic trans effect manifests itself with an increased labilization of the ligand trans to a given ligand and arises from an interplay between ground-state and transition-state effects. The term trans influence, on the other hand, is a ground-state effect only, describing the tendency of a given ligand to weaken the metal–ligand bond trans to itself. Herein, we will use the term “trans effect” to describe *both* the kinetic and the thermodynamic properties, whereas the term “trans influence” will refer *only* to thermodynamic properties. We will describe how these trans effects strongly impact the chemistry of the commonly encountered cyclometalated (N,C) Au(III) complexes, a class of complexes we have studied for more than a decade. We found that the outcome of reactions like alkylation, arylation, and alkynylation as well as halide metathesis are dictated by the different trans influence of the two termini of the chelating tpy ligand in (tpy)Au(OAc^F^)_2_ (tpy = 2-(*p*-tolyl)pyridine, OAc^F^ = OCOCF_3_, tpy-*C* > tpy-*N*). There is a strong preference for high trans influence ligands to end up trans to tpy-*N*, whereas the lower trans influence ligands end up trans to tpy-*C*. Taking advantage of these preferences, tailor-made (N,C)Au(III) complexes could be prepared. For the functionalization of alkenes at (tpy)Au(OAc^F^)_2_, the higher trans effect of tpy-*C* would suggest that the coordination site trans to tpy-*C* would be kinetically more available than the one trans to tpy-*N*. However, due to the thermodynamic preference of having the σ-bonded ligand, resulting from the nucleophilic addition to alkenes, trans to tpy-*N*, functionalization of alkenes was only observed trans to tpy-*N*. However, for a catalytic process, the reaction should happen trans to tpy-*C*, as was observed for the trifluoroacetoxylation of acetylene. When functionalizing acetylene in the coordination site trans to tpy-*N*, protolytic cleavage of the Au–C(vinyl) bond to release the product did not occur at all, whereas trans to tpy-*C* protolytic cleavage of the Au–C(vinyl) bond occurred readily, in agreement with the higher trans influence of tpy-*C* over tpy-*N*. The large impact of the trans effects in Au(III) complexes is finally exemplified with the synthesis of [(tpy)Au(π-allyl)]^+^[NTf_2_]^−^, which resulted in a highly asymmetric π + σ bonding of the allyl moiety. Here, the bonding is such that the most thermodynamically favorable situation is achieved, with the carbon trans to tpy-*N* bonded in a σ-fashion and the π-allyl double bond being coordinated trans to tpy-*C*.

## Key References

- Langseth, E.; Görbitz, C. H.; Heyn, R. H.; Tilset, M. Versatile Methods for Preparation of New Cyclometalated Gold(III) Complexes. *Organometallics***2012**, *31*, 6567–6571.^1^*Preparation of (N,C) mono- and dialkylated and arylated Au(III) complexes dictated by the trans influence of the chelating ligand*.
- Langseth, E.; Nova, A.; Tråseth, E. A.; Rise, F.; Øien, S.; Heyn, R. H.; Tilset, M. A gold exchange: A mechanistic study of a reversible, formal ethylene insertion into a gold(III)–oxygen bond. *J. Am. Chem. Soc*. **2014**, *136*, 10104–10115.^2^*Stoichiometric functionalization of ethylene in the coordination site trans to N of the chelating ligand.*
- Holmsen, M. S. M.; Nova, A. Balcells, D.; Langseth; E.; Øien-Ødegaard, S.; Heyn, R. H.; Tilset, M.; Laurenczy, G. Trans-Mutation at Gold(III): A mechanistic study of a catalytic acetylene functionalization via a double insertion pathway. *ACS Catal*. **2017**, *7*, 5023–5034.^3^*A stoichiometric functionalization of acetylene was obtained trans to N of the chelating ligand. In stark contrast, a catalytic functionalization of acetylene was achieved by utilizing the coordination site trans to C*.
- Holmsen, M. S. M.; Nova, A.; Øien-Ødegaard, S.; Heyn, R. H.; Tilset, M. A Highly Asymmetric Gold(III) η^3^-Allyl Complex. *Angew. Chem., Int. Ed.***2020**, *59*, 1516–1520.^4^*A highly asymmetric Au(III) π-allyl complex was obtained due to the electronically asymmetrical (N,C) ligand*.

## Introduction

Gold(III) complexes are generally stabilized by chelating, often bidentate ligands resulting from cyclometalation, and usually exhibit the expected square planar geometry.^5,6^ The nature of the chelate termini profoundly influences the properties of the two coordination sites that are localized trans to the chelate. Much of gold(III) catalysis occurs by binding and transforming substrate molecules at these two sites, and therefore, an appreciation of the trans effects of the chelating ancillary ligands is of utmost importance.

The “trans effect”, a term coined by Ilya Ilich Chernyaev in 1926,^7^ has earned itself a prominent position in the development and understanding of the structure and reactivity in coordination chemistry over the course of more than a century.^8−10^ The trans effect is an electronic effect most clearly noted in square planar complexes, and for a given ligand, it manifests itself with an increased labilization of the ligands trans to itself.^11^ Historically, the term trans effect has been applied to the effect of a coordinated group on the kinetics of ligand substitution reactions occurring trans to itself. This kinetic effect must arise from an interplay of ground-state and transition-state effects. On the other hand, the term “trans influence” has been used to describe the tendency of a given ligand to weaken specifically the metal–ligand bond trans to itself.^12^ This is a ground-state effect which will affect ground-state properties, i.e., bond distances, bond strengths, and spectroscopic properties. In the following, depending on the context, we will use the terminology “trans effect” to refer to both the thermodynamic and the kinetic effects, whereas the term “trans influence” will refer specifically to thermodynamic properties. The ranking of ligands according to decreasing kinetic trans effect at Pt(II) has been summarized as follows

CO, CN^–^, C_2_H_4,_ H^–^, CH_3_^**–**^ > PR_3_, NO_2_^–^, SCN^–^, I^–^ > Br^–^ > Cl^–^ > NH_3_ > OH^–^ > NO_3_^–^ > H_2_O^13,14^

CO, CN^–^, C_2_H_4_ > PR_3__,_ H^–^ > CH_3_^**–**^ > C_6_H_5_^–^, NO_2_^–^, I^–^, SCN^–^ > Br^–^, Cl^–^ > Py, NH_3_, OH^–^, H_2_O^15^

More or less subtle deviations from these sequences are often seen.^16−18^ As can be seen from the series above, good σ-donors and π-acceptors lead to large trans effects. It is important to keep in mind that the series is an empirical and approximate ranking, and in real situations, differences should be expected as one changes the metal, the charge, and the nature of the other ligands. All of these factors will have an impact on the orbitals for the metal and the ligands and hence the bonding between them. This together with sterics are mainly responsible for the trans effects.^15^ The paucity of quantitative studies of trans effects in Au(III) chemistry has been recently pointed out.^19^

Au(III) chemistry has currently undergone rapid development, and Au(III) complexes find applications in areas as diverse as catalysis, materials science, and medicinal uses.^19−23^ The rapid development of the field has been promoted by the availability of chelating (κ^2^) and pincer (κ^3^) ligands that serve to stabilize complexes of Au(III).^5,6,19^ Such chelating ligands show a great deal of structural variation and include combinations of neutral (L-type; commonly N or P donors) and formally anionic (X-type; typically aryl-*C* donors available by arene cyclometalation) ligands. The most commonly encountered systems for Au(III) κ^2^ chelates are of the types (N,N), (C,C), and (N,C). For unsymmetrical chelates, cyclometalated (N,C) systems are by far dominant and usually contain the phenylpyridine structural motif. Despite the prominent role of phosphines in transition-metal chemistry, cyclometalated (P,C) Au(III) complexes remain less explored than their (N,C) counterparts, but over recent years, their occurrence has increased noticeably. This topic was recently reviewed by Bourissou and co-workers.^24^

**Figure 1:**
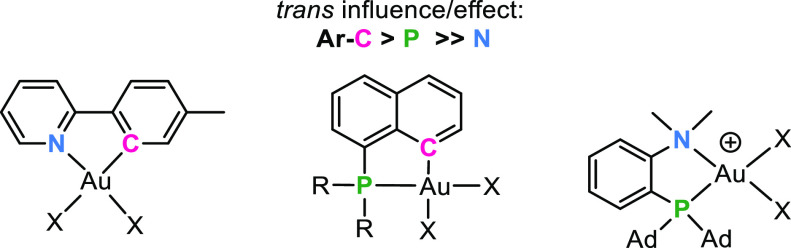
Generic representation of some representative Au(III) complexes bearing electronically unsymmetrical bidentate ligands, such as (N,C), (P,C), and (P,N) ligands. Ad = adamantyl.

From the trans effect series given above, it will be evident that a (N,C) chelate based on the phenylpyridine core might offer a square planar environment in which the two coordination sites that are trans relative to the two chelate sites will be electronically very different, which should be manifested in structures as well as reactivity patterns. On the other hand, analogous (P,C) systems are electronically more symmetrical on the basis of the more similar trans effects of the P and C termini of the chelate (Figure 1).

Herein, the main focus will be on cyclometalated (N,C) Au(III) complexes of the type Au(tpy)X_2_ (tpy = 2-(*p*-tolyl)pyridine) which have been thoroughly studied in our group (Figure 1). In these complexes, there are two distinctly different coordination sites available, one trans to tpy-*C* and one trans to tpy-*N*. It will be shown that the reactivity of these complexes is strongly dictated by the trans effects of the ancillary ligand, with tpy-*C* having a greater trans effect than tpy-*N*. The effect of the electronic asymmetry of the chelating ligand in reactions such as alkylations, arylations, and alkynylations together with ligand substitution reactions, nucleophilic additions, and protonolysis of Au–C bonds will be discussed. Insight into the consequences of the trans effects helps to further develop and improve Au(III) catalysis. Finally, the effect of the ancillary ligand will be briefly discussed for the newly discovered class of Au(III) π-allyl complexes. When appropriate, the properties of these (N,C) chelated complexes will be contrasted with the more recently reported (P,C) and (N,P) systems (Figure 1).

## Structural Manifestation of the Trans Influence

The relative thermodynamic trans influence of various ligands at a metal center can be assessed by several methods, the most common arguably being the comparison of relevant metric data obtained by single-crystal X-ray diffraction analyses. The method is particularly useful when metrics are available for unsymmetrically chelated complexes bearing identical groups in the two different trans positions. Table 1 lists some relevant metric parameters for selected representative (N,C), (P,C), and (P,N) Au(III) complexes depicted as structures **1a**–**d**.^25^ Comparisons of the two Au–X bond lengths trans to the termini of the (N,C) chelate in complexes **1a** show that the Au–X bonds that are trans to tpy-*C* are always significantly longer than those that are trans to tpy-*N* (by ca. 0.06–0.13 Å for the selected complexes), demonstrating that tpy-*C* has a consistently higher trans influence than tpy-*N*. Furthermore, by comparing relevant metric data in (P,C) complexes **1b** we find that the Au–X bond trans to naphthyl-*C* is always longer than the Au–X bond trans to naphthyl-*P* (by ca. 0.02–0.07 Å), indicating that naphthyl-*C* has a higher trans influence than naphthyl-*P*. Finally, in (P,N) complexes **1c** and **1d**, we observe that the Au–Cl and Au–C bonds trans to aryl-*P* are longer than those trans to N (by 0.08 and 0.05 Å, respectively), indicating that aryl-*P* has a higher trans influence than aryl-*N* in complexes **1c** and **1d**. Taken together, these data suggest a trans influence order of C > P ≫ N in these systems, in quite good agreement with the series presented in the Introduction.

**Table 1:**
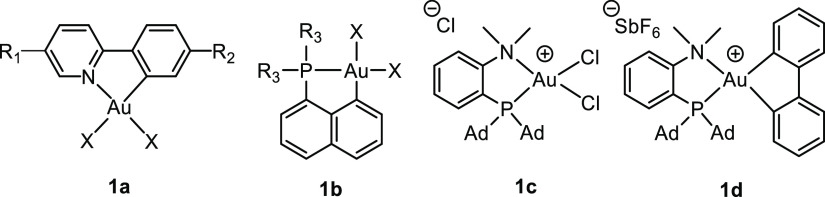
Selected Au–X Bond Distances [Angstroms] for (N,C), (P,C) , and (P,N) Au(III) Complexes **1a**–**d** Determined by X-ray Diffraction Analysis

		Au–X	Au–X	Au–X				
complex	X	trans to C	trans to P	trans to N	Au–C(aryl)	Au–N	Au–P	ref
**1a**, R_1_ = H, R_2_ = Me	Me	2.134(4)		2.038(4)	2.062(4)	2.130(3)		(1)
**1a**, R_1_ = H, R_2_ = Me	OAc^F^	2.111(5)		1.993(5)	1.995(7)	1.991(6)		(1)
**1a**, R_1_ = H, R_2_ = Me	–C≡C–Ph	2.031(4)		1.961(3)	2.042(3)	2.067(3)		(26)
**1a**, R_1_ = H, R_2_ = Me	–C≡C–TMS	2.07(2)		1.94(2)	2.048(2)	2.089(2)		(26)
**1a**, R_1_ = H, R_2_ = Me	–C≡C–H	2.07(2)		1.961(17)	2.026(17)	2.041(12)		(26)
**1a**, R_1_, R_2_ = CO_2_H	Cl	2.3613(6)		2.2713(6)	2.025(2)	2.041(2)		(27)
**1a**, R_1_, R_2_ = CO_2_H	Br	2.4745(5)		2.4128(5)	2.045(3)	2.066(3)		(28)
**1a**, R_1_, R_2_ = CO_2_H	I	2.6487(9)		2.5664(8)	2.05(1)	2.069(7)		(28)
**1b**, R = Ph	Cl	2.4263(14)	2.3538(16)		2.047(6)		2.2738(14)	(29)
**1b**, R = Ph	I	2.6621(3)	2.6288(3)		2.070(4)		2.284(1)	(29)
**1b**, R = *i*Pr	Me	2.117(5)	2.082(5)		2.101(4)		2.3161(11)	(30)
**1b**, R = *i*Pr	OAc^F^	2.105(3)	2.089(2)		2.004(3)		2.262(1)	(31)
**1c**			2.3521(12)	2.2727(13)		2.104(4)	2.3054(11)	(32)
**1d**			2.101(3)	2.048(3)		2.207(2)	2.4188(6)	(33)

Obviously, the X ligands at the two nonchelate coordination sites conversely exert their trans influences on the chelate atoms. From the available Au–C(aryl), Au–P, and Au–N data above, the relative trans influences of the X groups seem to decrease in the order Me > alkynyl ≥ I, Br, Cl > OAc^F^, in quite good agreement with anticipations based on the trans effect series presented in the Introduction.

## Preparative Au(III) Chemistry Dictated by the Trans Influence

**Scheme 1:**
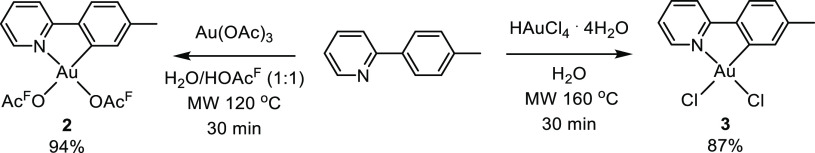
Microwave-Assisted Synthesis of Complexes **2** and **3**(1,34)

Easy access to the appropriate Au(III) complexes is of high importance to further develop the fundamental chemistry of Au(III) and gold catalysis. Therefore, a convenient and simple microwave-assisted synthesis giving access to the two key complexes Au(tpy)(OAc^F^)_2_ (**2**, OAc^F^ = OCOCF_3_) and Au(tpy)Cl_2_ (**3**) was developed (Scheme 1).^1,34^ Later, the synthesis of complex **2** was generalized to include a wide variety of electron-withdrawing substituents (CO_2_H, CO_2_Et, CF_3_, F, C_6_F_5_, and NO_2_) and electron-donating substituents (Me, OMe) on the phenylpyridine backbone, allowing for the fine-tuning of the electronic and steric properties of the ancillary ligand.^28,35^

**Scheme 2:**
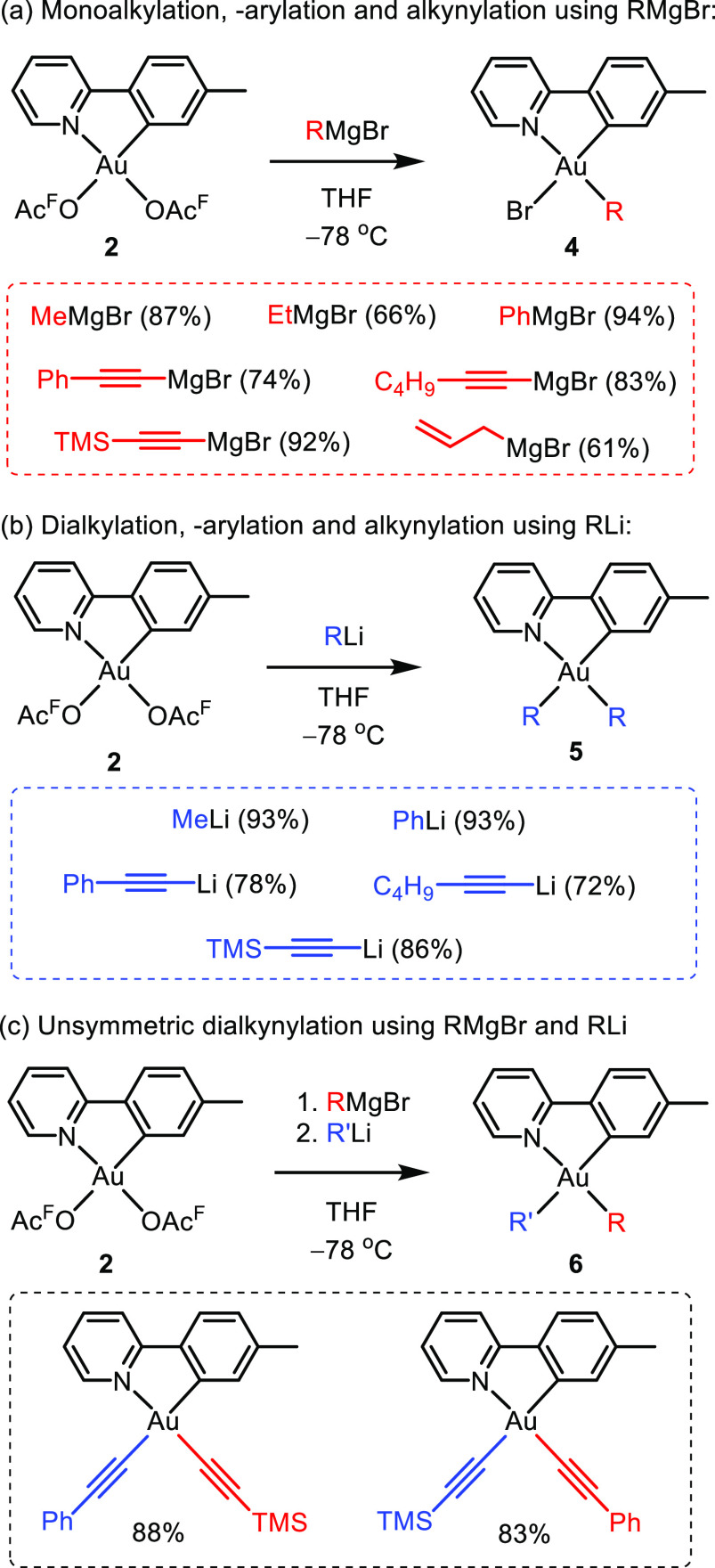
Reactivity of **2** toward RMgBr and RLi

Complex **2**, with the two labile OAc^F^ ligands offering two available coordination sites, was of special interest to us, and the possibilities for further derivatization of this complex were studied. Based on the concept of the trans effect, one would expect the OAc^F^ ligand trans to tpy-*C* to be kinetically more prone to undergo substitution than the one trans to tpy-*N*. This was supported experimentally by the ^19^F NMR resonance of the OAc^F^ ligand trans to tpy-*C* being significantly broadened, due to dynamic ligand exchange processes, in coordinating solvents such as CD_3_CN and HOAc-*d*_4_ compared to the resonance of the OAc^F^ ligand trans to tpy-*N*.^2^ However, based on the higher trans influence of tpy-*C* compared to tpy-*N*, the coordination of alkyl ligands should be thermodynamically preferred in the coordination site trans to tpy-*N*. Consistently, the stereoselective alkylation of complex **2** with Grignard reagents RMgX (R = Me, Et) could be achieved, furnishing the monoalkylated complexes Au(tpy)BrR **4** (Scheme 2a) with the alkyl group exclusively trans to tpy-*N* and Br trans to tpy-*C*, leading to the thermodynamic product.^1,4^ The reaction could also be extended to arylations (R = Ph) and alkynylations (R = C≡C–Ph, C≡C–C_4_H_9_, C≡C–TMS, Scheme 2a).^1,26^ When lithium reagents RLi were used instead of Grignard reagents RMgX, dialkylation, diarylation, and dialkynylation of complex **2** could be achieved (Scheme 2b) leading to complexes **5**.^1,26^ Finally, when the two methods in Scheme 2a and 2b were combined by reacting **2** first with RMgBr and then with R′Li, unsymmetrically alkynylated Au(III) complexes **6** could be selectively obtained by design (Scheme 2c).^26^

**Scheme 3:**
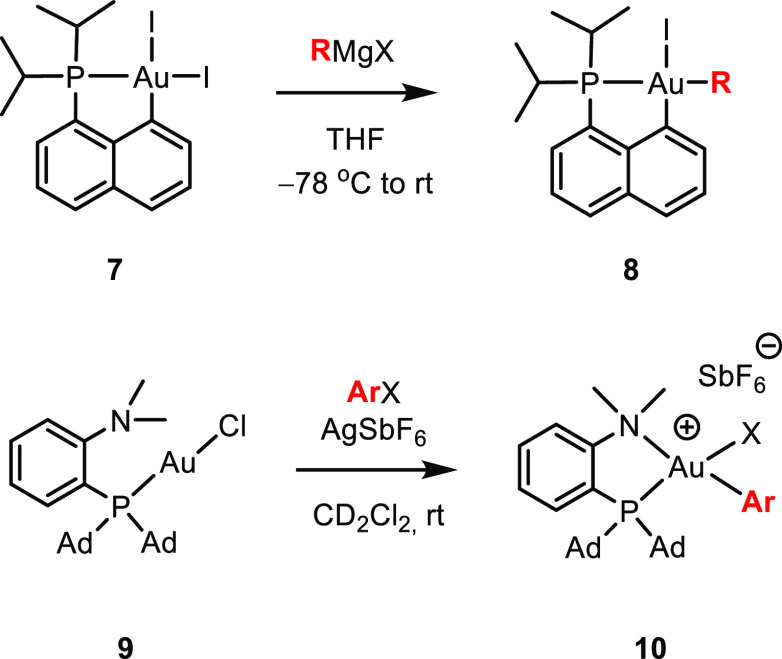
Monoalkylation and Monoarylation of (P,C) and (P,N) Au Complexes **7** and **9** R = alkyl, aryl, and allyl.

Stereoselective monoalkylation of Au(III) complexes bearing electronically unsymmetrical chelating ligands has also been achieved by Bourissou and co-workers utilizing the same strategy on (P,C)AuI_2_ complex **7** (Scheme 3, top).^30,36−38^ However, if an excess of Grignard reagent was utilized, alkylation both trans to P and trans to C was observed. Another approach developed by the same group takes advantage of the hemilabile MeDalphos ligand in Au(I) complex **9** to achieve oxidative addition of aryl halides (Scheme 3).^33^ In both cases, the most favorable arrangement with the alkyl or aryl group trans to P (complex **8**) or N (complex **10**) was observed.

**Scheme 4:**
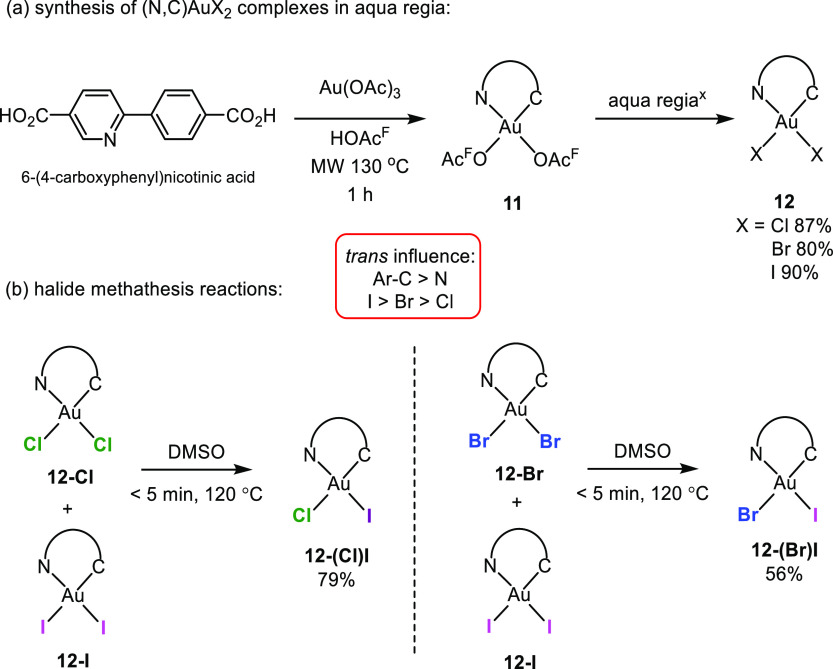
(a) Synthesis of (N,C)AuX_2_ Complexes **12** (X = Cl, Br, I) by Microwave Heating Followed by Aqua Regia^x^ Treatment; (b) Halide Metathesis Reactions of Complexes **12-Cl**, **12-Br**, and **12-I** Aqua regia^x^ = 3HX:HNO_3_; X = Cl, Br, I).

The synthesis and purification of several (N,C)Au(III) dihalide complexes is also possible by using aqua regia.^28,39,40^ This synthetic methodology was particularly useful when using 6-(4-carboxyphenyl)nicotinic acid instead of tpy, which offered preparative and workup challenges. The use of this ligand was motivated by our desire to covalently incorporate carboxy-functionalized, cyclometalated complexes into UiO-67^41^ type metal organic frameworks (MOFs).^27^ Heating a mixture of 6-(4-carboxyphenyl)nicotinic acid and Au(OAc)_3_ in HOAc^F^ in a microwave reactor led to the formation of bis(trifluoroacetate) complex **11** (Scheme 4a).^28^ Subsequent treatment of complex **11** with aqua regia^*x*^ (aqua regia^*x*^ = 3HX:HNO_3_; X = Cl, Br, I)^42^ furnished the corresponding dihalide complexes **12** in good yields (Scheme 4a). As already mentioned, the Au–C(aryl) and Au–N(pyr) bond distances in these dihalide complexes suggest rather similar, yet slightly decreasing, trans influences in the order I > Br > Cl. Interestingly, when complexes **12-Cl** and **12-I** were mixed in a 1:1 ratio in DMSO, a clean transformation into only one single, detectable species **12-(Cl)I** with I trans to N was observed (Scheme 4b). The mixed halide species **12-(Cl)I**, which has I trans to N and Cl trans to C, circumvents the undesirable situation of positioning the two stronger trans influence ligands (I and C) opposite to each other in the square plane. A similar and anticipated halide redistribution behavior was seen when complexes **12-Br** and **12-I** were mixed to furnish **12-(Br)I**, again with I trans to pyridine-*N*. This stereochemical outcome suggests thermodynamic control of the reactions, in full agreement with the relative trans influence series of the halides (I > Br > Cl).^43^

## Functionalization of Alkenes and Alkynes at Au(III)

Having established a convenient synthesis for complex **2**, with the two potentially available coordination sites trans to tpy-*C* and tpy-*N*, we were interested in studying its reactivity toward small, unsaturated molecules, such as ethylene and acetylene.

**Scheme 5:**
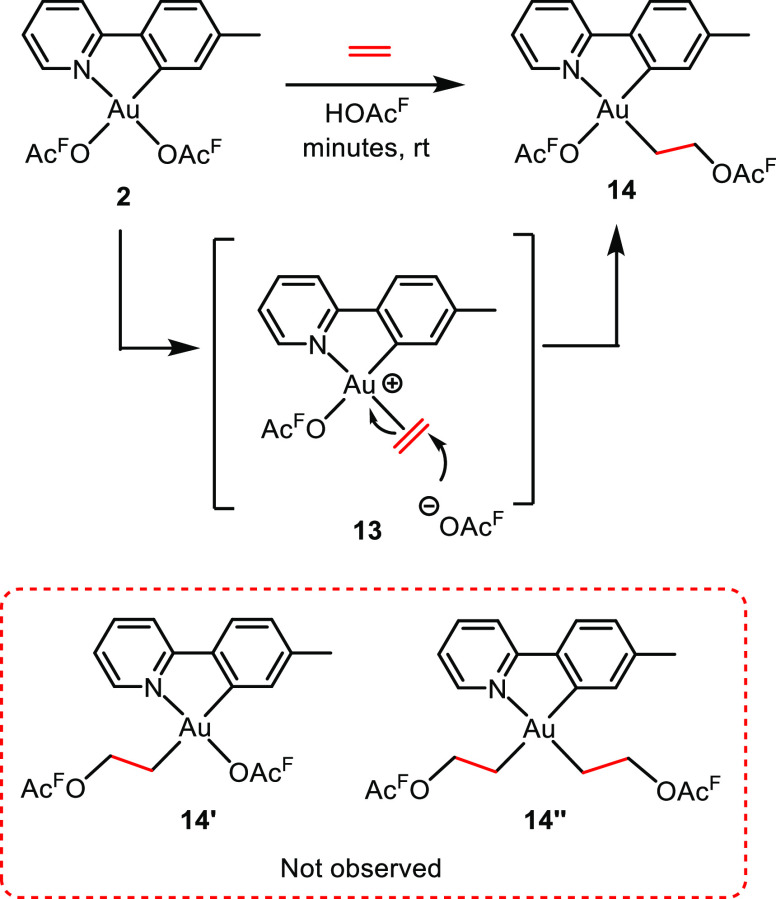
Formal Insertion of Ethylene into the Au–O Bond Trans to tpy-N in Complex **2** Furnishing Complex **14** with the C(sp^3^) Ligand Trans to tpy-N

In 2014, we reported the formal insertion of ethylene into the Au–O bond trans to tpy-*N* in complex **2** (Scheme 5).^2^ The reaction proceeds via a substitution of the trifluoroacetate ligand trans to tpy-*N* by ethylene followed by intermolecular nucleophilic addition of trifluoroacetate to complex **13** furnishing complex **14**. Remarkably, the formal insertion only occurred in the position trans to tpy-*N*; therefore, neither complex **14′** nor complex **14′′** was observed (Scheme 5). DFT calculations showed that even though the coordination site trans to tpy-*C* is kinetically available (Δ*G*^‡^ = 16.3 kcal mol^–1^ for the associative substitution of OAc^F^ with ethylene trans to tpy-*C*), the formation of the corresponding product **14′**, with two high trans influence ligands trans to each other, is precluded because the overall reaction is endergonic (Δ*G* = 13.7 kcal mol^–1^ with respect to **2**). Furthermore, DFT calculations showed that the nucleophilic addition of trifluoroacetate to ethylene, trans to tpy-*C* and to tpy-*N*, would both be reversible, explaining why the thermodynamic product was the only obtained product.

**Scheme 6:**
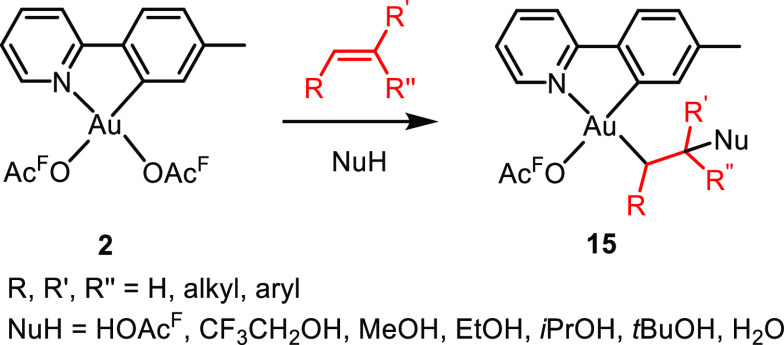
Nucleophilic Addition to Alkenes at Complex **2** Furnishing Complexes **15** (**14** when R, R′, R′′ = H and NuH = HOAc^F^)

The nucleophilic addition to ethylene could be extended to include a wide variety of alkenes and nucleophiles leading to complexes **15**.^44^ The general scope of the reaction is summarized in Scheme 6. As observed for the reaction of **2** with ethylene in HOAc^F^, no nucleophilic addition was observed trans to tpy-*C*, again demonstrating the strong preference for these high trans influence C(sp^3^) ligands to bind trans to tpy-*N*. Furthermore, the nucleophilic additions always occurred at the most substituted end of the double bond, i.e., in a Markovnikov manner.

**Scheme 7:**
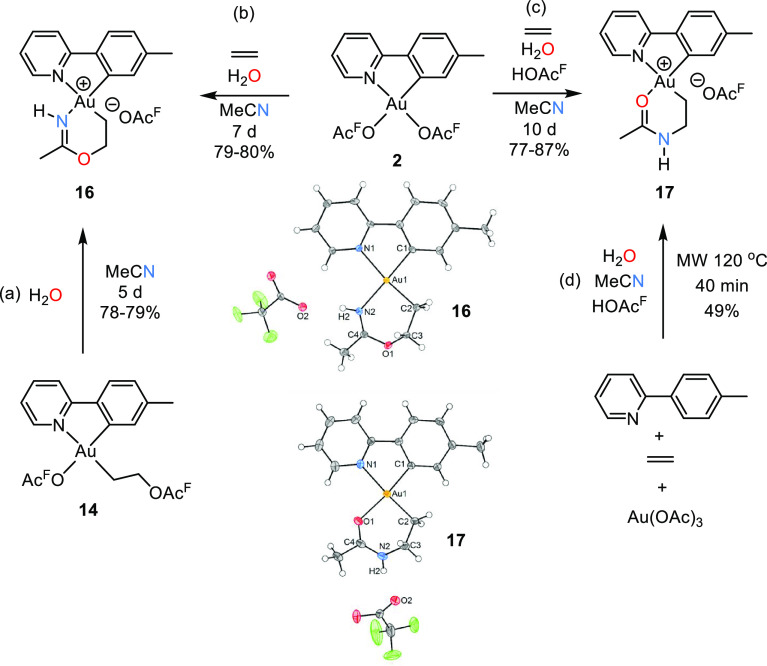
Metallacycle Formation at Au(III) Formation of (N,C) metallacycle **16** from **14** (a) and **2** (b). Formation of (O,C) metallacycle **17** from **2** (c), one-pot microwave synthesis from Au(OAc)_3_ (d), and ORTEP plots of **16** and **17** with ellipsoids at 50%.

Although no formal insertion was observed in the coordination site trans to tpy-*C* in the reactions of complex **2** with alkenes (Scheme 6), the coordination site trans to tpy-*C* is kinetically accessible. Therefore, further functionalization of complex **14** by introducing a ligand with a lower trans influence could be a possibility. Indeed, we found that by stirring complex **14** in wet MeCN, cyclization occurred to form the metallacycle **16** (path a, Scheme 7).^45^ In complex **16**, a *N*-ligand, with a weaker trans influence, is located trans to tpy-*C*, which is a more favorable situation compared to that of **14′** and **14′′** (Scheme 5). The preparation of complex **16** could also be performed in a one-pot reaction, thereby incorporating the three building blocks ethylene, acetonitrile, and water into one gold complex in just one step, dictated by the relative trans influence of tpy-*C* and tpy-*N* (path b, Scheme 7).

Interestingly, by addition of HOAc^F^ to the reaction mixture (see Path b vs Path c in Scheme 7), the outcome of the reaction was quite different. Instead of the formation of the *N*-bonded metallacycle **16**, the *O*-bonded metallacycle **17** was formed. Complex **17** could also be prepared in a one-pot microwave procedure from Au(OAc)_3_, tpyH, ethylene, H_2_O, MeCN, and HOAc^F^ (Path d, Scheme 7), showing the remarkable preference for all of these readily available building blocks to assemble in one specific way guided by the trans influence of the ligands. DFT calculations on the formation of **16** and **17** suggest that both reactions follow similar mechanisms involving the consecutive addition of water and acetonitrile to intermediate **13** (Scheme 5) in a different order (see SI for details).

**Scheme 8:**
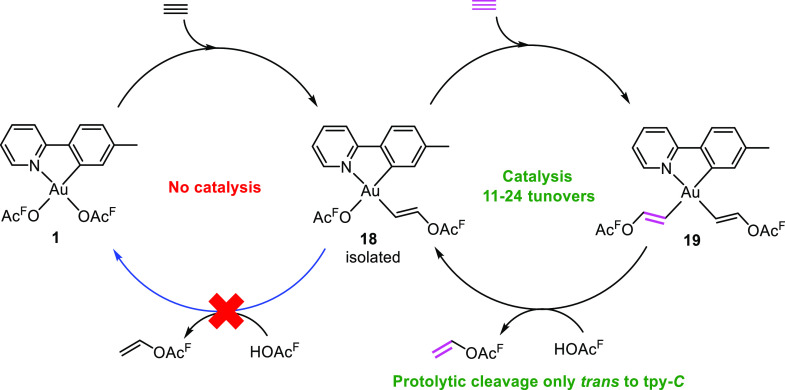
Catalytic Transformation of Acetylene with Complex **2** as Precatalyst and Complex **18** as the Active Catalyst

Unfortunately, we were not able to obtain a catalytic process for the reactions with alkenes at Au(III) as protolytic cleavage of the Au–C(sp^3^) bonds trans to tpy-*N* does not occur. As a consequence, we turned our focus to the reactivity of **2** with alkynes. To our delight, upon bubbling acetylene through a solution of complex **2** in HOAc^F^ at ambient temperature, catalytic trifluoroacetoxylation of acetylene was achieved (Scheme 8).^3^ Upon performing the reaction at 0 °C, we were able to isolate Au(III) vinyl complex **18** with the vinyl group trans to tpy-*N*, in agreement with the thermodynamic preference of having the high trans influence vinyl group trans to tpy-*N* (Δ*G* = −20.7 kcal mol^–1^ for **18**, whereas with the vinyl group trans to tpy*-C* Δ*G* = −4.9 kcal mol^–1^).^46^ However, upon dissolving complex **18** in HOAc^F^, no catalysis was observed and **18** remained stable in solution. The catalysis did not continue until acetylene was added to the reaction mixture. These results indicate that the Au–C(vinyl) bond in complex **18** does not undergo protolytic cleavage. This agreed with computations, which demonstrated that the free energy barrier for the protolytic cleavage of the Au–C(vinyl) bond in **18** is prohibitively high (Δ*G*^‡^ = 24.8 kcal mol^–1^) for a reaction occurring at room temperature. Instead, we discovered after meticulous mechanistic studies including deuterium-labeling experiments and DFT calculations that a second formal insertion of acetylene occurs, this time trans to tpy-*C*, forming Au(III) divinyl complex **19**. From complex **19**, the protolytic cleavage of the Au–C(vinyl) bond trans to tpy-*C* occurs readily (Δ*G*^‡^ = 14.3 kcal mol^–1^), leading to the release of vinyl trifluoroacetate and the regeneration of complex **18**. This agrees with the higher trans influence of tpy-*C* vs tpy-*N* as the Au–vinyl bond trans to tpy-*C* should be weaker and easier to protolytically cleave.

**Scheme 9:**
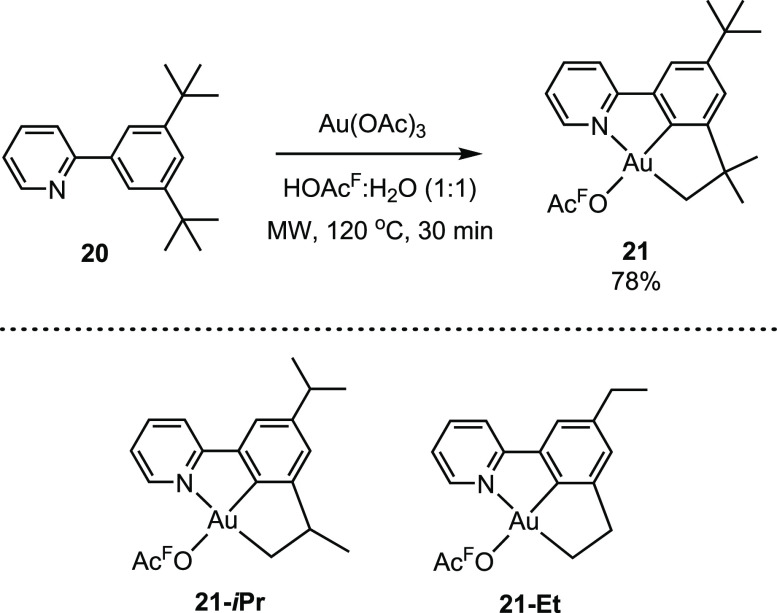
Synthesis of Au(III) Pincer Complexes **21** via C(sp^3^)–H and C(sp^2^)–H Activation

Based on the findings described above, we envisioned that a more direct way to promote reactivity in the position trans to tpy-*C* would be by using (N,C,C)-pincer complexes. The synthesis of (N,C,C) pincer complex **21** was successfully accomplished from Au(OAc)_3_ and **20** utilizing the same conditions as those used for the synthesis of complex **2** (Scheme 9).^47^ The formation of **21** occurs via C(sp^2^)–H activation, which is common for Au(III), and C(sp^3^)–H activation, which is scarcely reported for Au(III).^47^ Later, complexes **21**-***i*Pr** and **21-Et** were also prepared by a related protocol.^35^ Complex **21** was tested in the catalytic trifluoroacetoxylation of acetylene, and it was indeed shown to be an improved and significantly more robust catalyst for this transformation. Less than 10% decomposition of the catalyst was seen after 24 h of catalysis, in contrast to complete decomposition observed for the system using complex **2** as a precatalyst.^3,47^

**Scheme 10:**
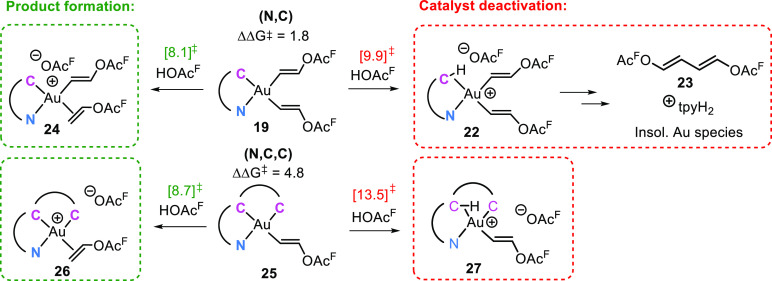
Ligand Protonation and Potential Decomposition Pathways for Complexes **19** and **25**; Free Energies in kcal mol^–1^

To rationalize the improved robustness of the catalytic systems using complex **21** rather than **2**, we consider the preference for the protolytic cleavage of the Au–C(vinyl) bond trans to aryl-*C* compared to processes that lead to catalyst deactivation in complexes **19** and **25** (Scheme 10). In **19** and **25**, there are two Au–C(sp^2^) bonds trans to each other that can be protolytically cleaved, leading to catalysis (Scheme 10, left) or catalyst decomposition (Scheme 10, right). In the case of **19**, a difference of less than 2 kcal mol^–1^ was found between the protolytic cleavage of the Au–C(vinyl) bond, leading to catalysis, and the protolytic cleavage of the Au–C(tpy) bond, leading to reductive elimination of the experimentally detected **23** and catalyst deactivation (Scheme 9).^47^ Indeed, after 24 h of catalysis with complex **2** as precatalyst, complete decomposition was observed, and no molecular gold complexes could be observed in solution by ^1^H NMR spectroscopy.^3^ In contrast, for **25**, the difference in the free energy barrier for the protolytic cleavage of the Au–vinyl bond compared to the Au–C(aryl) bond is ca. 5 kcal mol^–1^.^47^ Moreover, complex **27** resulting from the protolytic cleavage of the Au–C(aryl) bond is still a chelate, impeding its further decomposition via reductive elimination.

## Au(III) π-Allyl Complexes

**Scheme 11:**
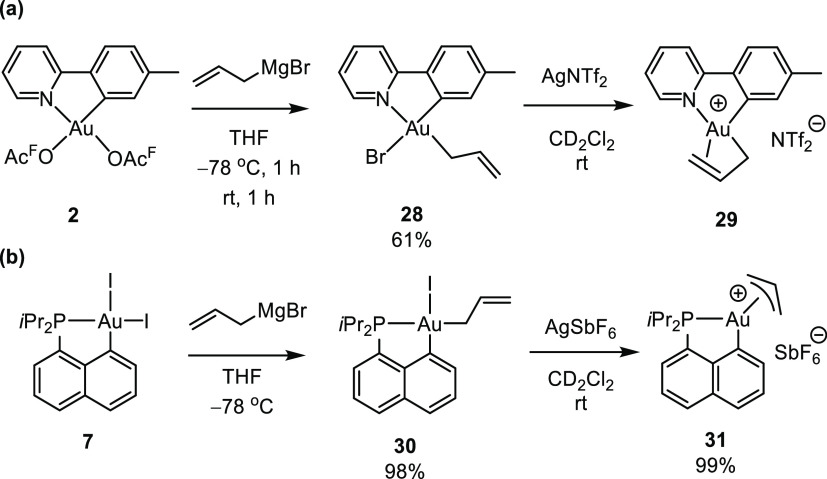
Synthesis of Cyclometalated Au(III) π-Allyl Complexes **29** and **31** upon Reaction of Their Corresponding σ-Allyl Complexes **28** and **30** with a Silver Salt

**Figure 2:**
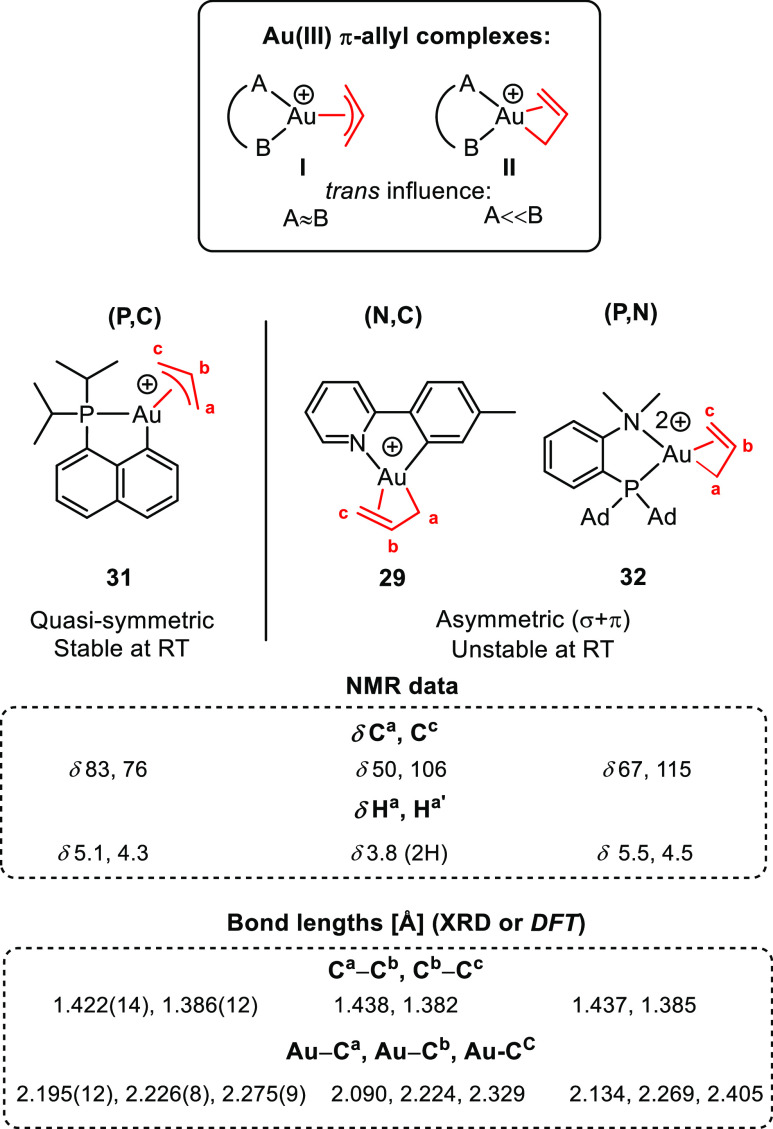
Au(III) π-allyl complexes reported by the Tilset group and the Bourissou group. Ad = adamantyl.

Despite transition-metal π-allyl complexes being thoroughly studied over the years, they were only very recently reported for gold.^48^ Two simultaneous reports on (N,C) and (P,C) Au(III) π-allyl complexes appeared in 2020 from the Bourissou group^36^ and our group^4^ (Scheme 11). Both groups utilized the same strategy for the synthesis of the π-allyl complexes: (i) allylation of complexes **2** and **7** furnished the σ-allyl complexes **28** and **30**, respectively, both with the thermodynamically preferred trans arrangement of the σ-allyl and the donor atom of the chelating ligand (N or P, respectively). (ii) Thereafter, the opening of a coordination site at gold upon reaction with a silver salt led to the formation of the corresponding Au(III) π-allyl complexes **29** and **31**. Following this, the Bourissou group reported that the (P,C) Au(III) π-allyl complex **31** undergoes nucleophilic attack of β-keto enolates leading to C–C coupling products as the final product.^38^ More recently, they reported the catalytic Au(I)/Au(III) allylation of indoles where the key intermediate was (P,N) Au(III) π-allyl complex **32** (Figure 2).^49^

Comparing the three types of characterized Au(III) π-allyl complexes depicted in Figure 2, it becomes evident that the ancillary ligand plays a remarkable role on the stability and symmetry of the π-allyl complexes.^4,36,49,50^ Whereas (P,C) Au(III) π-allyl complex **31** has a quasi-symmetric π-allyl ligand and is stable at room temperature, the (N,C) and (P,N) Au(III) π-allyl complexes **29** and **32** exhibit an asymmetric bonding of the π-allyl ligand (σ + π) and both complexes are unstable at room temperature.

The differences in bonding in complexes **29**, **31**, and **32** were evident from their NMR spectra, their respective bond distances, and the electronic structure analyses of the complexes.^4,36,49,50^ (i) The quasi-symmetric coordination of the π-allyl in **31** is illustrated by the equalization of the chemical shifts of the two terminal carbons C^a^ and C^c^, whereas in complexes **29** and **32** they are found at significantly different chemical shifts (δ 83/76 for **31** vs δ 50/106 and δ 67/115 for complexes **29** and **32**, respectively). (ii) The bond lengths for C^a^–C^b^ and C^b^–C^c^ in complex **31** (1.422(14) vs 1.386(12) Å, respectively) are similar, whereas for complexes **29** and **32**, they are much more different (1.438 vs 1.382 Å for **29** and 1.437 vs 1.385 Å for **32**). Moreover, in complexes **29** and **32**, C^a^ is much more tightly bound than C^b^ and C^c^, indicating a significant σ-character to the Au–C^a^ bond. (iii) When analyzing the bonding situation in complexes **29**, **31**, and **32** by the atoms-in-molecules (AIM) approach,^48^ bond critical points (BCP) are found between both the terminal carbons of the allyl moiety and the gold in complex **31**, whereas for complexes **29** and **32**, BCP is *only* found between gold and C^a^. Moreover, the electron densities and the Bader’s delocalization indexes found by AIM show a delocalized allyl moiety for **31**, whereas for **29** and **32** it shows a significant double-bond character for C^b^–C^c^. These striking differences can be attributed to the different electronic properties of the ancillary ligand, i.e., the difference in trans influence of the two ends of the (P,C), (N,C), and (P,N) chelates, where P and C in complex **31** are closer to each other than N and C in complex **29** and N and P in complex **32**. Complexes **29** and **32** are, therefore, best described as Lewis structure **II** (Figure 2, top) exhibiting a σ + π bonding with the high trans influence sp^3^ part of the π-allyl located trans to the lower trans influence end of the chelating ligand (the N atom). On the other hand, complex **31** is best described by structure **I** with a quasi-symmetric bonding of the allyl moiety.

The asymmetric σ + π bonding in complexes **29** and **32** may suggest observable fluxionality in these complexes. However, this was only observed for complex **29**. The dynamic behavior in complex **29** was evident from its ^1^H NMR spectrum; the two H^a^ are found as one resonance, in contrast to what is normally observed for π-allyl complexes (see Figure 2).^4^ This phenomenon indicates that double-bond decoordination followed by rotation of the AuCH_2_–CHCH_2_ bond and then recoordination occurs relatively fast on the NMR time scale, leading to coalesced resonances for the two H^a^. The fact that coalesced ^1^H NMR signals only were observed for the two H^a^ and not for the two H^c^ indicates that decoordination occurs selectively trans to tpy-*C* and not trans to tpy-*N* in accordance with the σ + π coordination of the π-allyl in complex **29** and the thermodynamic preference of having the σ-character trans to tpy-*N* and not trans to tpy-*C*.

**Figure 3:**
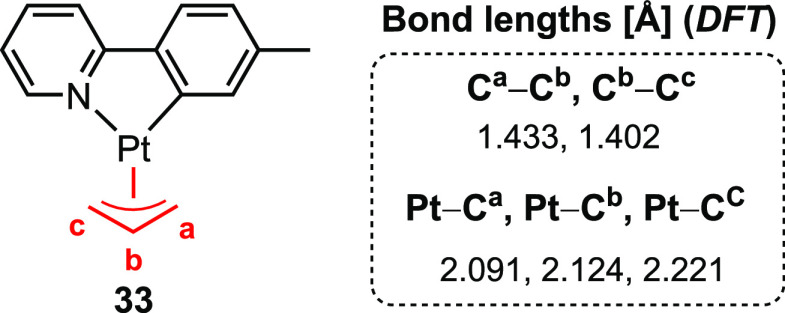
Computed bond lengths for (N,C) Pt(II) π-allyl complex **33**.

The structure of (N,C) Pt(II) π-allyl complex **33** was computed and compared to that of the closely related (N,C) Au(III) π-allyl complex **29** (Figure 3).^4^ Interestingly, the geometric data obtained for complex **33** shows a less asymmetrically bonded π-allyl than that of complex **29**. This result further suggests a more significant trans influence in square planar Au(III) complexes compared to other transition metal complexes. One might speculate whether the increased trans influence of Au vs Pt in the π-allyl systems may be due to increased covalent character in Au–ligand bonds (a consequence of the exceptionally high electronegativity of Au; Pauling electronegativity 2.54) compared to other transition metals, including Pt (2.28).^51^ This explanation agrees with the two models considered for the origin of the trans influence.^12^

## Summary and Outlook

The reactivity of cyclometalated (N,C) Au(III) complexes, like (tpy)Au(OAc^F^)_2_, is strongly dictated by the trans effects of the chelating ligand. For reactions like alkylation, arylation, and alkynylation together with functionalization of alkenes there is a strong selectivity for high trans influence C ligands to end up trans to tpy-*N*, whereas the lower trans influence ligands end up trans to tpy-*C*. This phenomenon was further demonstrated in halide metathesis reactions where mixing two Au(III) dihalide species led to the scrambling of the halides, furnishing the most thermodynamically favored arrangement with the higher trans influence halide trans to tpy-*N*. In the catalytic transformation of acetylene, nucleophilic addition to acetylene could occur trans to both tpy-*N* and tpy-*C*, but catalysis was only observed trans to tpy-*C* because only there was the Au–C(vinyl) bond weak enough to undergo protolytic cleavage. This knowledge was then used to construct an improved catalyst where the coordination site trans to tpy-*N* was blocked. The difference in trans influence was also shown to greatly affect the bonding of the π-allyl moiety in Au(III) π-allyl complexes; with electronically asymmetric (N,C) and (P,N) ligands, an asymmetric σ + π bonding of the allyl moiety was observed, whereas for the close to electronically symmetric (P,C) ligand, quasi-symmetric bonding of the allyl moiety was observed. The knowledge gained over the past decade on how the trans effects impact the chemistry of Au(III) complexes is important to better understand Au(III) catalysis and to facilitate the preparation of the relevant Au(III) complexes. By the use of the right ancillary ligand, the Au(III) complex in question may be tailor-made to fit to the desired application, which is of great importance both during catalysis and for the synthesis of important key Au(III) complexes.

## Supporting Information Available

Experimental details and characterization data for complex **17** together with computational details and proposed reaction mechanism for the formation of complexes **16** and **17** (PDF)
