# Supplementary material for: Cyclometalated (N,C) Au(III) Complexes: The Impact of Trans Effects on Their Synthesis, Structure, and Reactivity
Source: Acc Chem Res. 2023 Dec 5;56(24):3654–64. doi: 10.1021/acs.accounts.3c00595 (PMC10734256; doi:10.1021/acs.accounts.3c00595)
Supplement: Supplementary file 1 — ar3c00595_si_001.pdf [file ar3c00595_si_001.pdf]

Supporting Information

Cyclometalated (N,C) Au(III) complexes: the influence of *trans* effects on their synthesis, structure and reactivity

Marte Sofie Martinsen Holmsen, [a,b,]* *Ainara Nova,*[a,b,c,d] *and Mats Tilset* [a,b,c,]*

[a] *Department of Chemistry, University of Oslo, P.O. Box 1033 Blindern, N-0315 Oslo, Norway.*

[b] *Centre for Materials Science and Nanotechnology*, University of Oslo, P.O. Box 1126 Blindern, N-0316 Oslo, Norway

[c] *Hylleraas Centre for Quantum Molecular Sciences, Department of Chemistry, University of Oslo, P.O. Box 1033, Blindern, N-0315 Oslo, Norway.*

[d] *UiT-The Arctic University of Norway*, N-9037 Tromsø, Norway.

*Corresponding authors: m.s.m.holmsen@kjemi.uio.no, mats.tilset@kjemi.uio.no

Table of contents:

General procedures S2

Synthesis of complex **17** from complex **2** S3

One-pot synthesis of complex **17** from $Au(OAc)_3$ S4

Crystallographic structure determination of complex **17** S5

NMR spectra of complex **17** S7

Computational details S15

References S17

General procedures

Complex **2** was prepared by a previously reported procedure.[1] $Au(OAc)_3$ was purchased from ABCR. CH_2Cl_2 and MeCN were purified using a MB SPS-800 solvent purifying system from MBraun. CD_2Cl_2 was dried over 3Å molecular sieves. Ethylene 3.5 was purchased from Hydro Gas. Distilled water was used in all reactions and workups. All other reagents and solvents were used as received. All reactions were performed in air. As a precaution, all syntheses were performed in the absence of light. NMR spectra were obtained on DPX200, AVI600, and AVII600 instruments at ambient temperature. ^{1}H and ^{13}C NMR spectra have been references relative to the residual solvent signals (CD_2Cl_2: $\delta(^{1}H)$ 5.34, $\delta(^{13}C)$ 53.84). The ^{15}N NMR chemical shifts have been calibrated using $MeNO_2$ as an external standard at 0 ppm by adding a closed capillary containing $MeNO_2$ to the NMR sample of interest. ^{19}F NMR has been referenced relative to $CFCl_3$ by using C_6F_6 (−164.9 ppm with respect to $CFCl_3$ at 0 ppm) as an internal standard by adding 0.5-1 µL C_6F_6 to the NMR sample of interest. The peaks in the ^{1}H NMR spectra were assigned by the aid of 2D NMR techniques such as COSY, HSQC, HMBC, and NOESY. Mass spectra (ESI) were obtained on a Bruker maXis II ETD spectrometer by Osamu Sekiguchi (University of Oslo).

Synthesis of complex 17 from complex 2

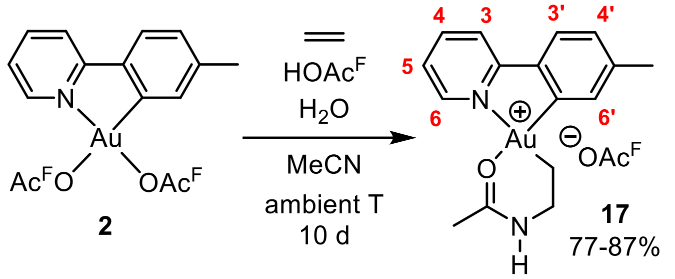

Complex **2** (51.8 mg, 0.0876 mmol, 1.0 equiv.) was dissolved in acetonitrile (4 ml). HOAc^F (0.50 mL, 6.5 mmol, 74 equiv.) and water (10 µL, 0.55 mmol, 6.3 equiv.) was added. Ethylene was bubbled through the solution for two minutes, and the flask was sealed with a glass stopper. The reaction mixture was stirred at ambient temperature in the absence of light for 10 days. The volatiles were removed under reduced pressure and the remaining solid was dissolved in CH_2Cl_2 and filtered. The solvent was removed under reduced pressure yielding **17** (43.0 mg, 0.0762 mmol, 87%) as a white solid. NB: It is difficult and sometimes impossible to get rid of all the excess HOAc^F, therefore the yield might be overestimated. For NMR spectra see Figure S2-S16. Broadening of the NMR resonances are observed, however, addition of HOAc^F to the NMR sample leads to less broadening of the resonances.

^{1}H NMR (600 MHz, CD_2Cl_2): δ 11.8 (br. s, 1H, N**H**), 8.75 (br. d, 1H, *J* = 3.8 Hz, **H^6**), 8.11 (ddd, 1H, *J* = 8.0, 7.7, 1.6 Hz, **H^4**), 7.99 (d, 1H, *J* = 8.2 Hz, **H^3**), 7.70 (d, 1H, *J* = 7.9 Hz, **H$^{3'}$**), 7.60 (ddd, 1H, *J* = 7.5, 5.4, 0.8 Hz, **H^5**), 7.24-7.25 (m, 2H, **H$^{4'}$** and **H$^{6'}$**), 3.47 (m, 2H, NC**H$_2$**), 2.69 (br. m, 2H, AuC**H$_2$**), 2.44 (s, 3H, ArC**H$_3$**), 2.40 (br. s, 3H, OCC**H$_3$**N).

^{1}H NMR (600 MHz, CD_2Cl_2 with *ca.* 0.7 equiv HOAc^F added): δ 9.47 (br. s, 1H, N**H**), 8.76 (d, 1H, *J* = 5.2 Hz, **H^6**), 8.13 (ddd, 1H, *J* = 8.0, 7.7, 1.5 Hz, **H^4**), 8.00 (d, 1H, *J* = 8.1 Hz, **H^3**), 7.71 (d, 1H, *J* = 7.9 Hz, **H$^{3'}$**), 7.62 (ddd, 1H, *J* = 6.9, 6.1, 0.8 Hz, **H^5**), 7.26 (d, 1H, *J* = 7.9, **H$^{4'}$**), 7.20 (s, 1H, **H$^{6'}$**), 3.46 (m, 2H, NC**H$_2$**), 2.71 (m, 2H, AuC**H$_2$**), 2.44 (s, 3H, ArC**H$_3$**), 2.42 (s, 3H, OCC**H$_3$**N).

^{19}F NMR (188 MHz, CD_2Cl_2): δ –78.2.

^{13}C NMR (151 MHz, CD_2Cl_2): δ 175 (br, O**C**CH$_3$NH), 161.0 (br. q, *J* = *ca.* 35 Hz, O**C**OCF$_3$), 160.6, 145.7, 143.1, 142.4, 140.7, 135.8, 132.0, 129.6, 126.1, 124.3, 120.4, 38.1 (br., HN**C**H$_2$), 34.0 (br., Au**C**H$_2$), 22.5, 21.9. Several of the peaks are broadened and the resonance at δ 175 is barely visible due to broadening. OCO**C**F$_3$ was not observed.

^{15}N{^{1}H} NMR (600 MHz, CD_2Cl_2 with *ca.* 0.7 equiv HOAc^F added): δ –122 (**N**$_{tpy}$), –242 (**N**H).

MS (ESI, MeCN): *m/z* (rel.%): 451 ([M–OAc^F]$^+$, 100).
HRMS (ESI, MeCN): Found 451.1080; calcd. for $C_{16}H_{18}AuN_2O$ 451.1079.

One-pot synthesis of complex 17 from $Au(OAc)_3$

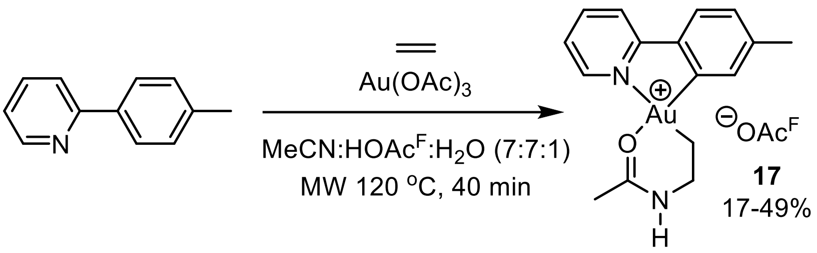

$Au(OAc)_3$ (196.2 mg, 0.5244 mmol, 1.04 equiv) was added to a microwave vessel. A mixture of water (1 mL), $HOAc^F$ (7 mL), MeCN (7 mL), and tpyH (100 µL, 0.504 mmol, 1.00 equiv) was added. Ethylene was bubbled through the mixture for 1 minute, the vessel was sealed and the reaction mixture was heated in a microwave at 120 °C for 40 min. After the reaction, a brownish mixture was obtained. CH_2Cl_2 (15 mL) was added, and the solution was filtered. The mixture was transferred to a separation funnel and water (10 mL) was added. The organic phase was collected and the water phase was extracted with CH_2Cl_2 (2 x 10 mL). The combined organic phases were washed with water (3 x 10 mL), dried over Na_2SO_4, and filtered. The solvent was removed under reduced pressure furnishing a dark oil. The dark oil was redissolved in CH_2Cl_2 and filtered through celite furnishing a yellow solution. CH_2Cl_2 was again removed under reduced pressure and the remaining yellow oil was crystallized from CH_2Cl_2/pentane furnishing **17** (139.6 mg, 0.2474 mmol, 49%) as a white solid. NB: It is difficult and sometimes impossible to get rid of all the excess $HOAc^F$, therefore the yield might be overestimated.

Crystallographic structure determination of complex 17

Crystals suitable for X-ray diffraction analysis of complex **17** were obtained by placing a small vial containing **17** dissolved in CH_2Cl_2 in a capped larger vial containing pentane in a refrigerator (*ca*. 10 °C).

Single crystal diffraction data for complex **17** was acquired on a Bruker D8 Venture equipped with a Photon 100 detector and using Mo Kα radiation (λ = 0.71073 Å) from an Incoatec iµS microsource. Data reduction was performed with the Bruker Apex3 Suite, [2] the structure was solved with ShelXT[3] and refined with ShelXL. [4,2] Olex2 was used as user interface.[5] The cif files were edited with enCIFer v1.4, [6] and molecular graphics were produced with Diamond v4.4.0.[7] The crystallographic structure determination was performed by Dr. Sigurd Øien-Ødegaard (University of Oslo).

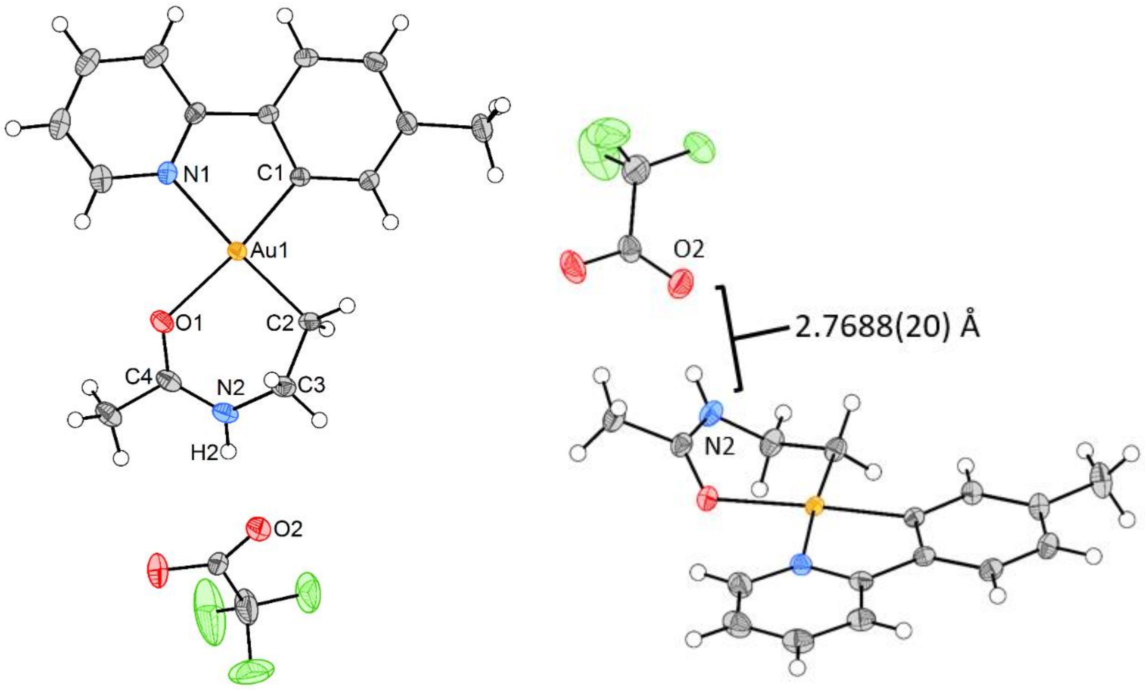

Figure S1. ORTEP plots of complex **17** with 50% ellipsoids. $^{-}OAc^{F}$ is disordered (not shown). Selected bond distances [Å] and angles [°]: Au1-N1, 2.1129(14); Au1-C1, 1.9921(16); Au1-O1, 2.0985(12); Au1-C2, 2.0361(16); C2-C3, 1.521(2); C3-N2, 1.466(2); N2-C4, 1.307(2); C4-O1, 1.278(2); O2···N2, 2.7688(20); N1-Au1-C1, 81.73(6); C2-Au1-O1, 92.37(6).

Table S1. Crystal and refinement data for complex **17**.

	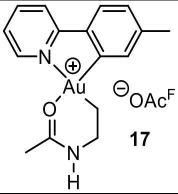 **17**
**Crystal data**	
CCDC number	2295959
Identification code	MSH-454
Chemical formula	$C_{16}H_{18}AuN_2O{\cdot}C_2F_3O_2$
M_r	564.31
Crystal system, space group	Monoclinic, *C*2/*c*
Temperature (K)	100
a, *b*, *c* (Å)	12.8485(7), 21.8433(12), 14.0317(11)
α, β, γ (°)	90, 109.346(1), 90
V ($Å^3$)	3715.7 (4)
Z	8
Radiation type	Mo *K*α
μ (mm^{-1})	7.97
Crystal size (mm)	0.15 x 0.09 x 0.08
**Data Collection**	
Diffractometer	Bruker D8 Venture, CMOS detector diffractometer
Absorption correction	Multi-scan SADABS-2014/5 (Bruker,2014/5) was used for absorption correction. wR2(int) was 0.0704 before and 0.0379 after correction. The Ratio of minimum to maximum transmission is 0.7652. The λ/2 correction factor is Not present.
T_{min}, T_{max}	0.572, 0.747
No. of measured, independent and observed [I > 2σ(I)] reflections	46041, 9013, 7801
R_{int}	0.026
(sin θ/λ)max ($Å^{-1}$)	0.835
**Refinement**	
$R[F^2 > 2\sigma(F^2)]$, $wR(F^2)$, S	0.017, 0.053, 1.19
No. of reflections	9013
No. of parameters	264
No. of restraints	0
H-atom treatment	H-atom parameters constrained
	$w = 1/[\sigma^2(F_o^2) + (0.0281P)^2 + 0.5356P]$ where $P = (F_o^2 + 2F_c^2)/3$
$\Delta\rho_{max}$, $\Delta\rho_{min}$ (e $Å^{-3}$)	1.00, −1.96

NMR spectra of complex 17

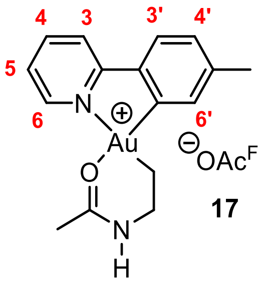

17

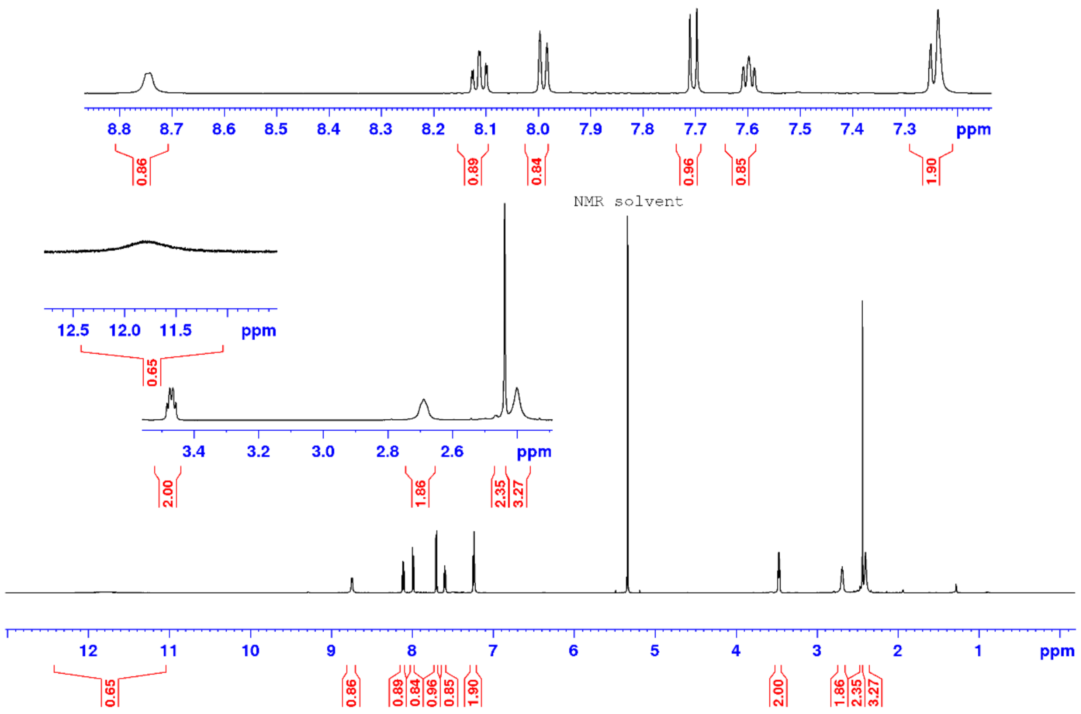

Figure S2. ^{1}H NMR (600 MHz, CD_2Cl_2) spectrum of complex **17**. Several of the resonances are broadened.

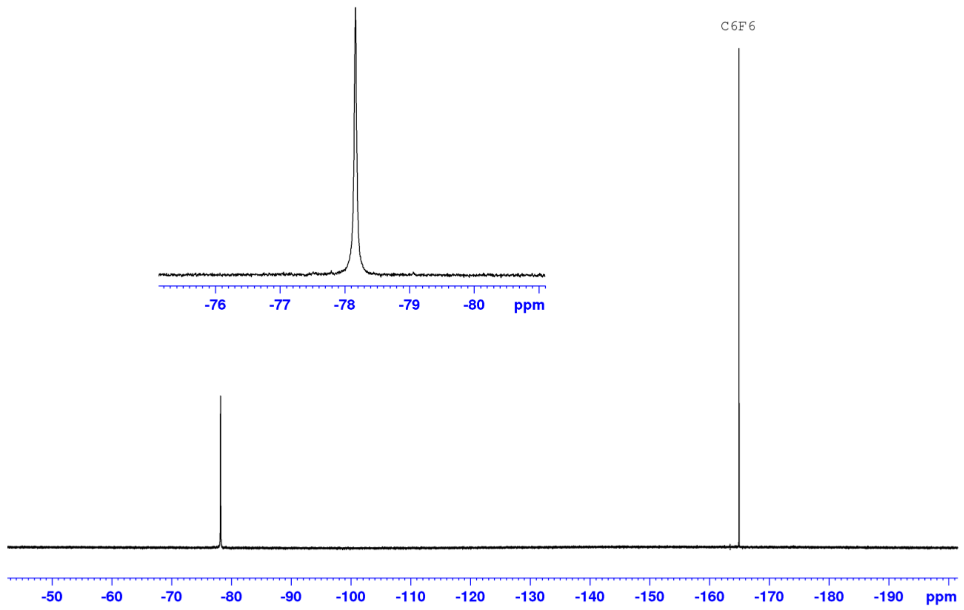

Figure S3. ^{19}F NMR (188 MHz, CD_2Cl_2) spectrum of complex **17**.

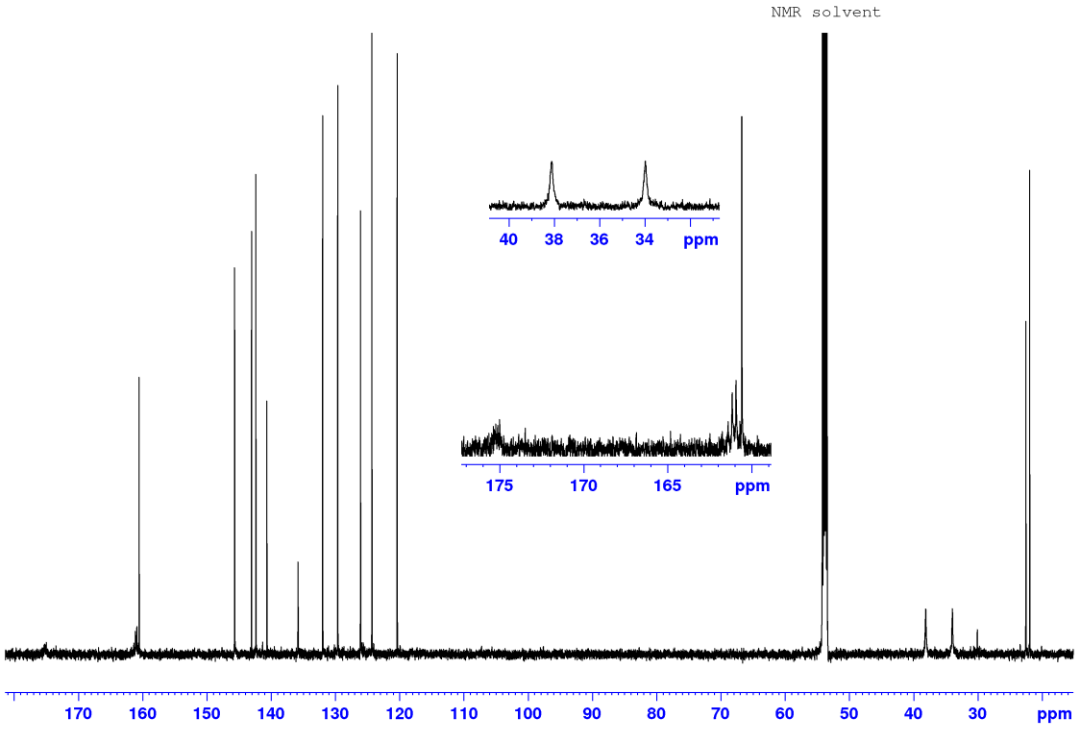

Figure S4. ^{13}C NMR (151 MHz, CD_2Cl_2, ns = 5120, d1 = 6 s) spectrum of complex **17**. Several of the resonances are broadened, especially those of O**C**CH_3 (δ 175), O**C**OCF_3 (δ 161.0), HN**C**H_2 (δ 38.1), and Au**C**H_2 (δ 34.0, see insets).

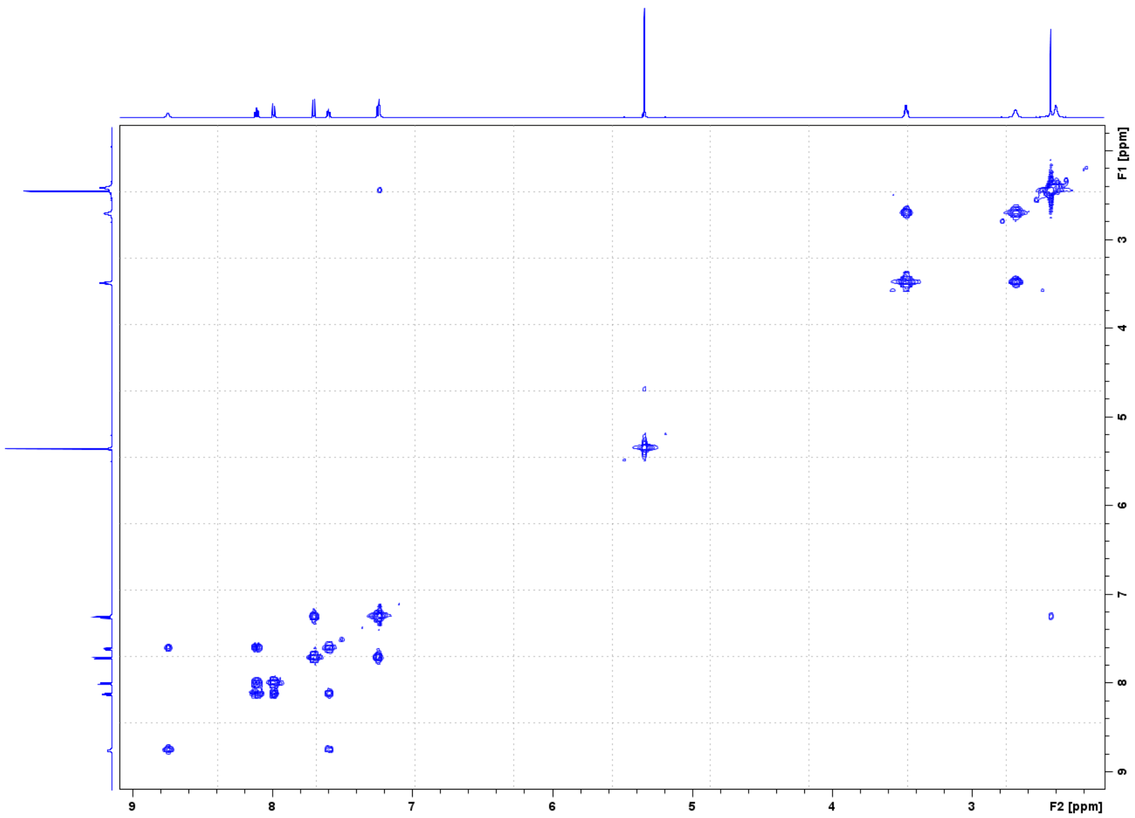

Figure S5. COSY (600 MHz, CD_2Cl_2) spectrum of complex **17**.

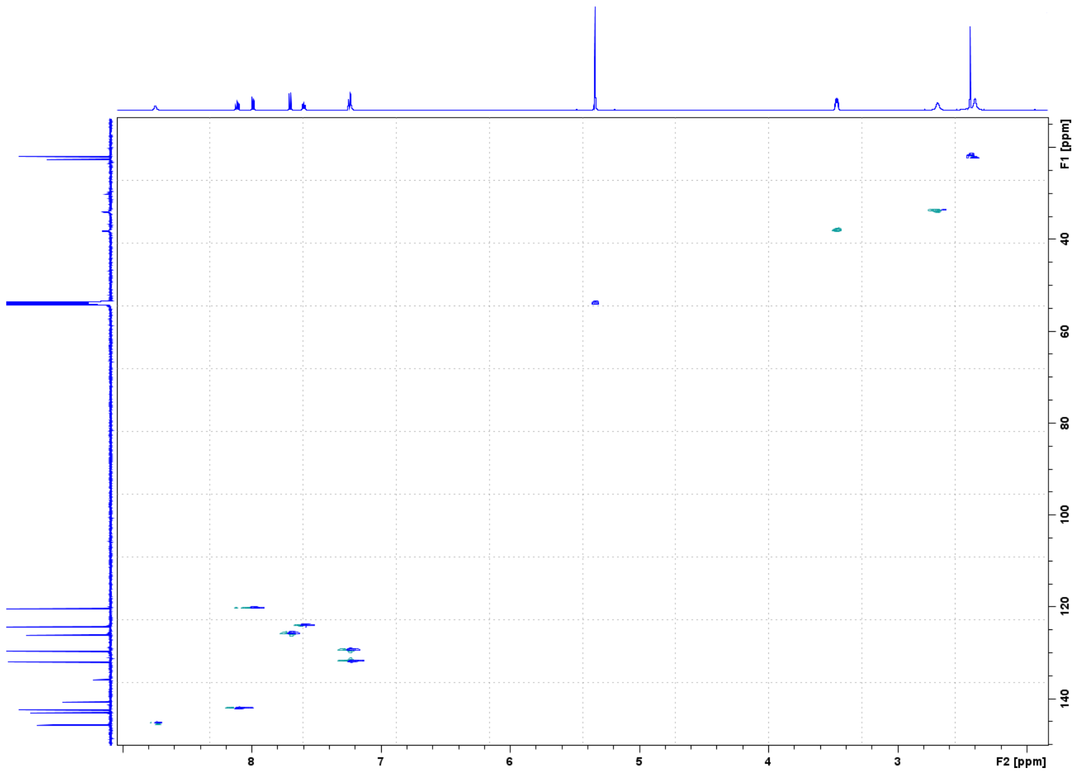

Figure S6. HSQC (600 MHz, CD_2Cl_2) spectrum of complex **17**.

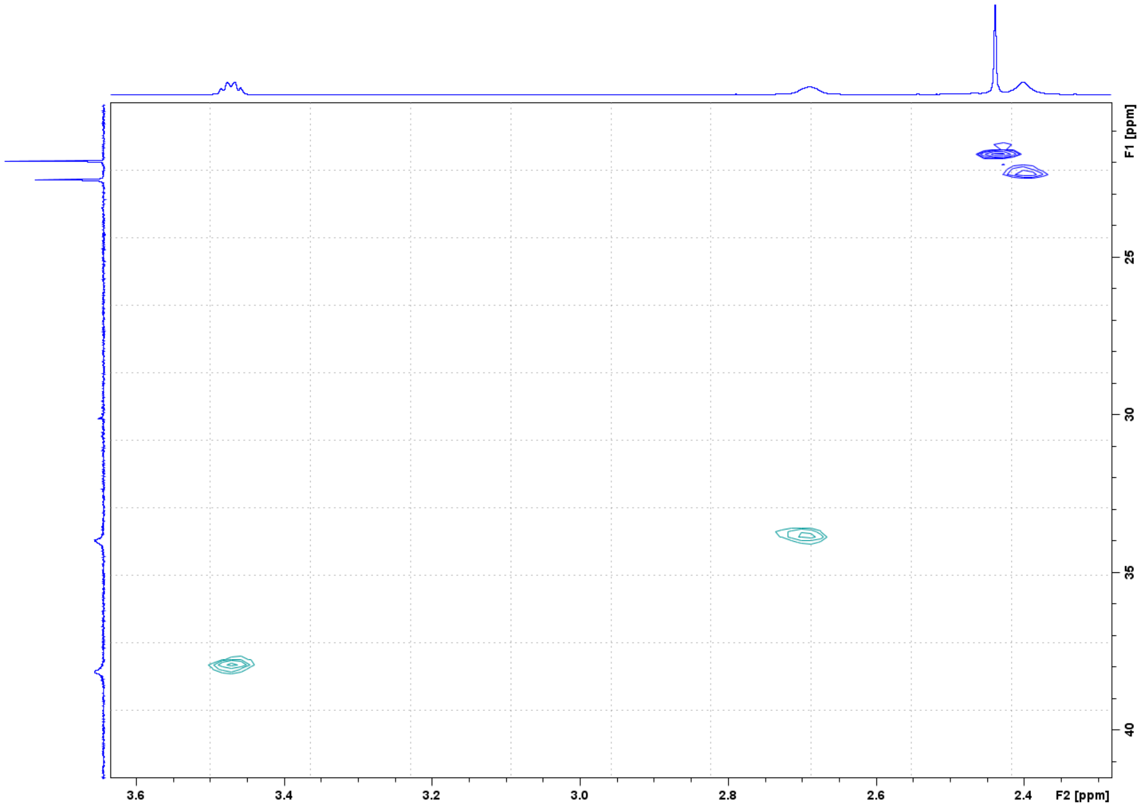

Figure S7. HSQC (600 MHz, CD_2Cl_2) spectrum of complex **17**. Close up view on the aliphatic region.

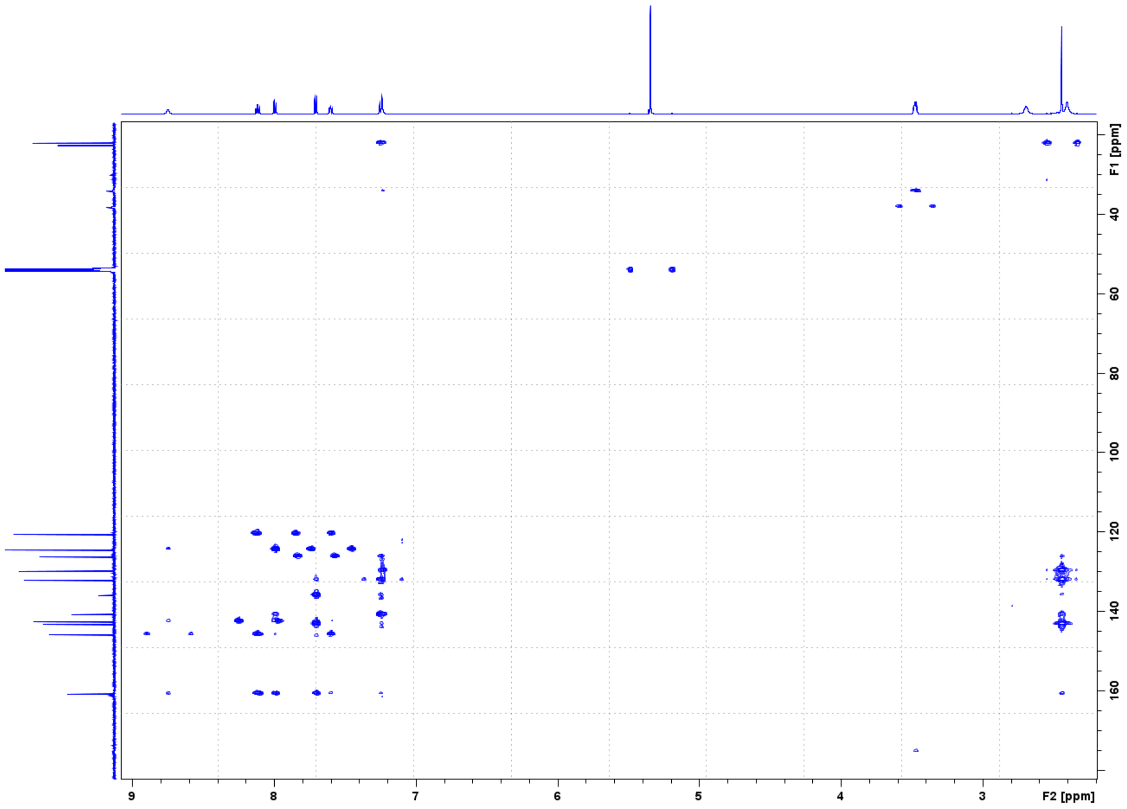

Figure S8. HMBC (600 MHz, CD_2Cl_2) spectrum of complex **17**.

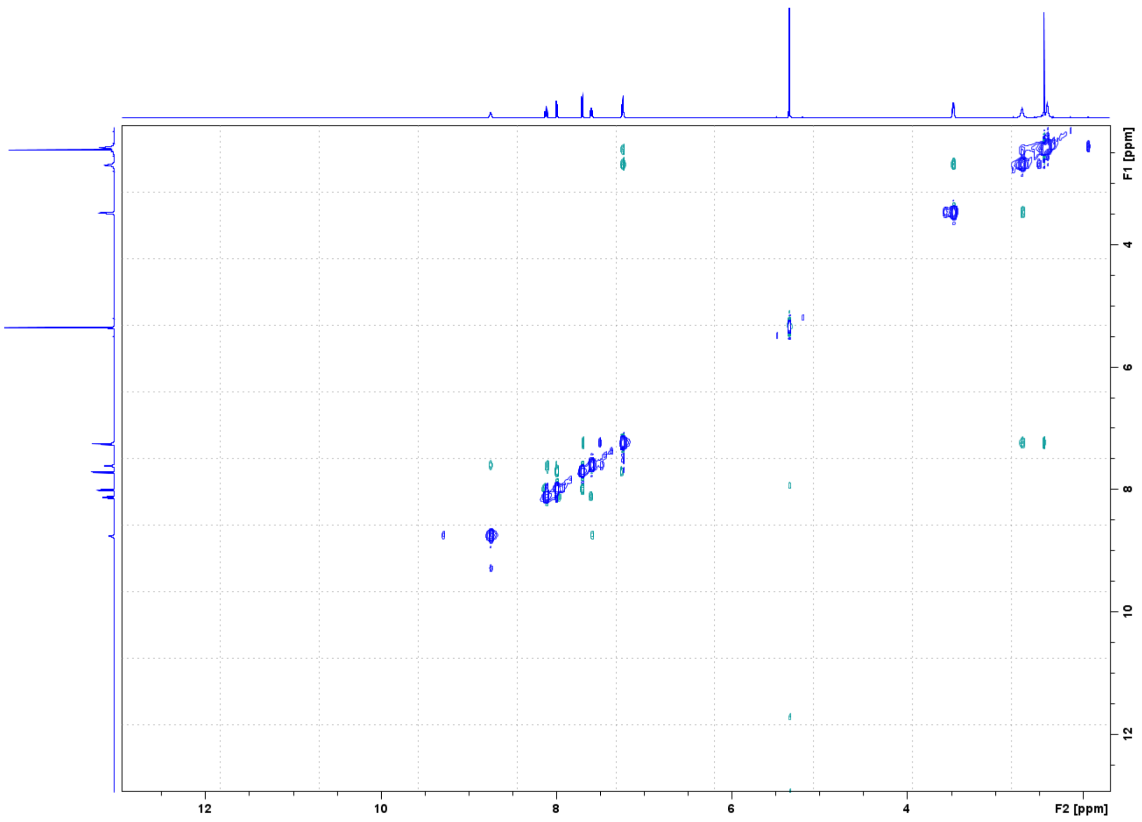

Figure S9. NOESY (600 MHz, CD_2Cl_2, mixing time = 1 s) spectrum of Au(III) complex **17**. Peaks due to what is probably chemical exchange are observed.

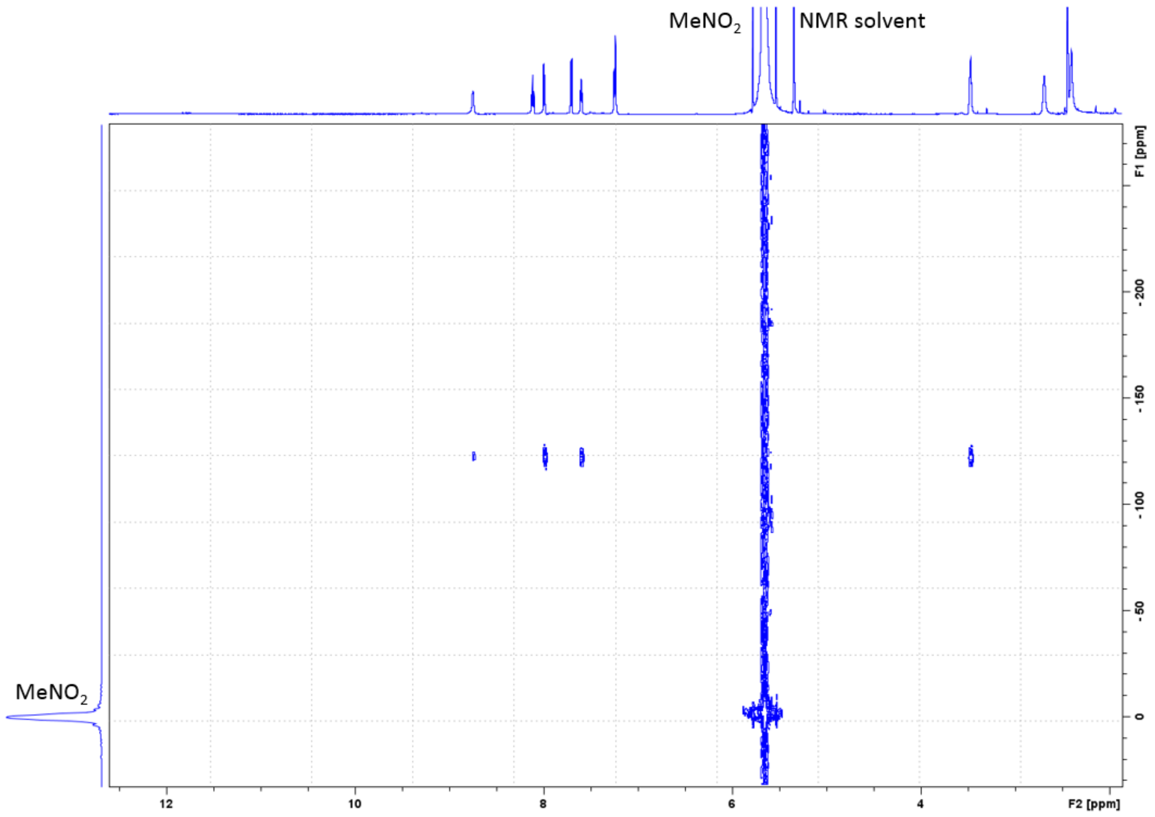

Figure S10. ^{1}H-^{15}N HMBC (600 MHz, CD_2Cl_2) spectrum of **17**. The resonance of the metallacycle-*NH* is not observed.

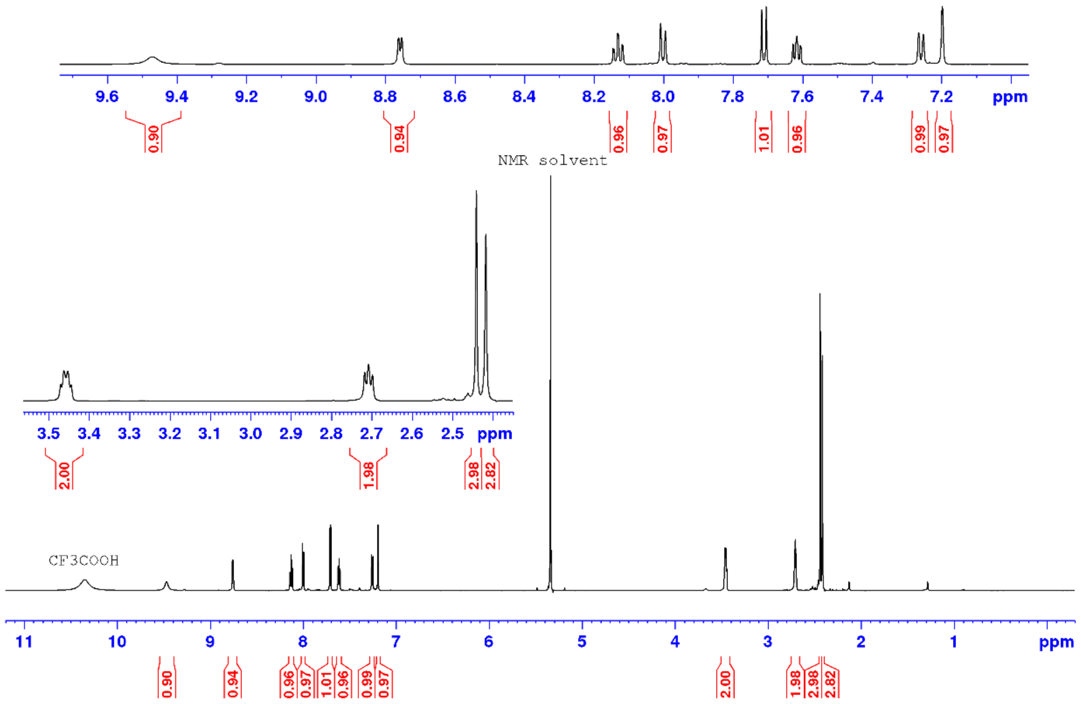

Figure S11. ^{1}H NMR (600 MHz, CD_2Cl_2) spectrum of complex **17** with *ca.* 0.7 equiv of CF_3COOH added.

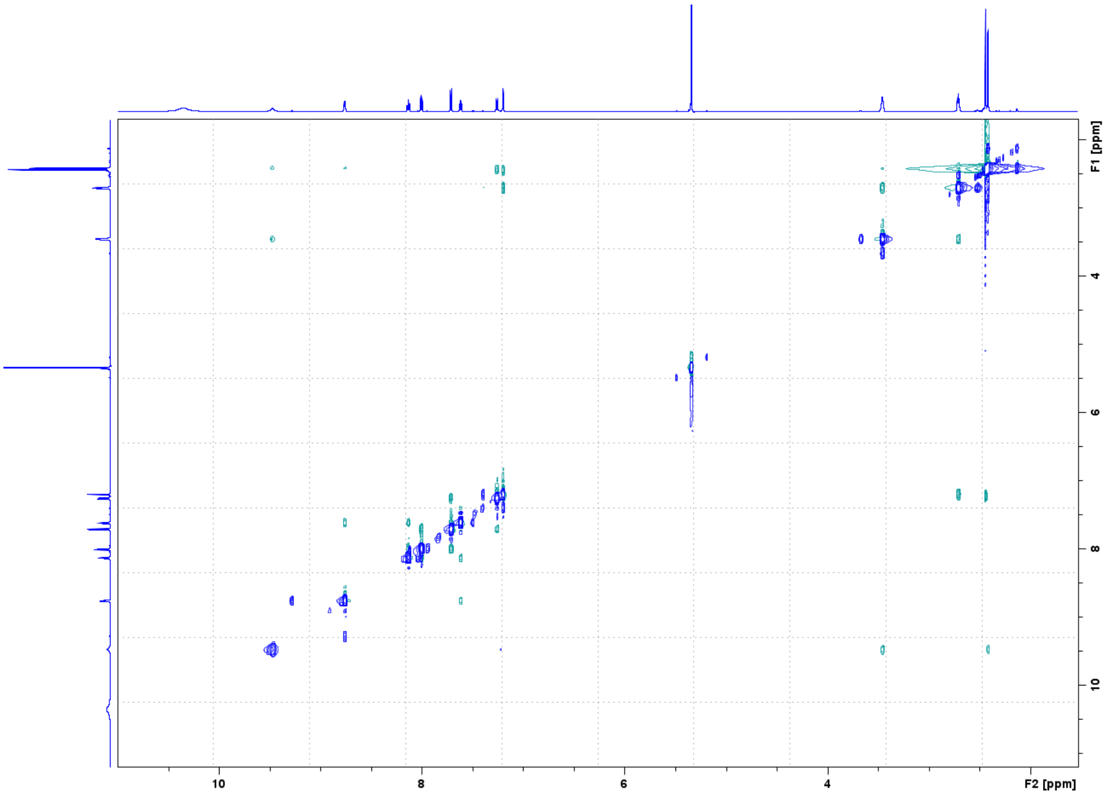

Figure S12. NOESY (600 MHz, CD_2Cl_2, mixing time = 1 s) spectrum of complex **17** with *ca.* 0.7 equiv HOAcF added. The peak at *ca.* δ 10.4 is due to excess HOAcF. Peaks due to what is probably chemical exchange are observed.

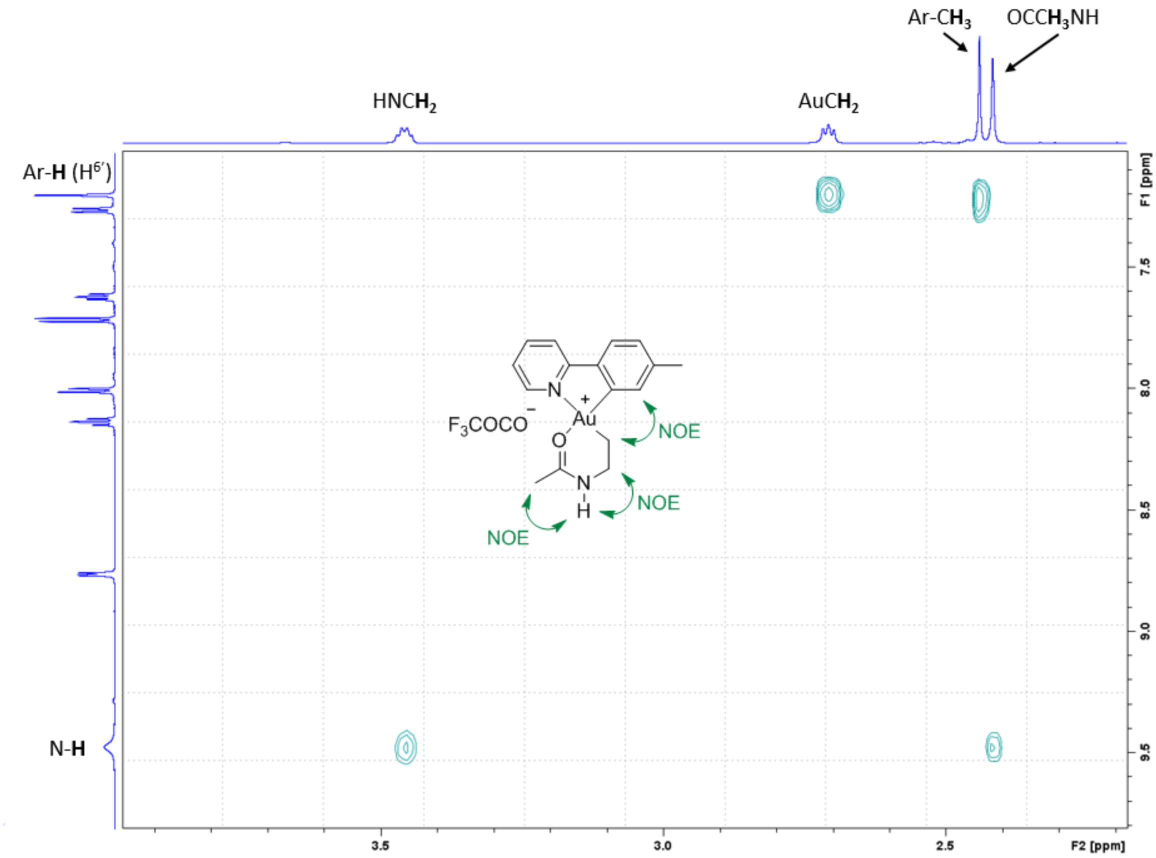

Figure S13. NOESY (600 MHz, CD_2Cl_2, mixing time = 1 s) spectrum of **17** with *ca*. 0.7 equiv HOAcF added. Close up view on the NOE between AuC**H₂** and H6′, N**H** and NHC**H₂**, and N**H** and OCC**H₃**NH.

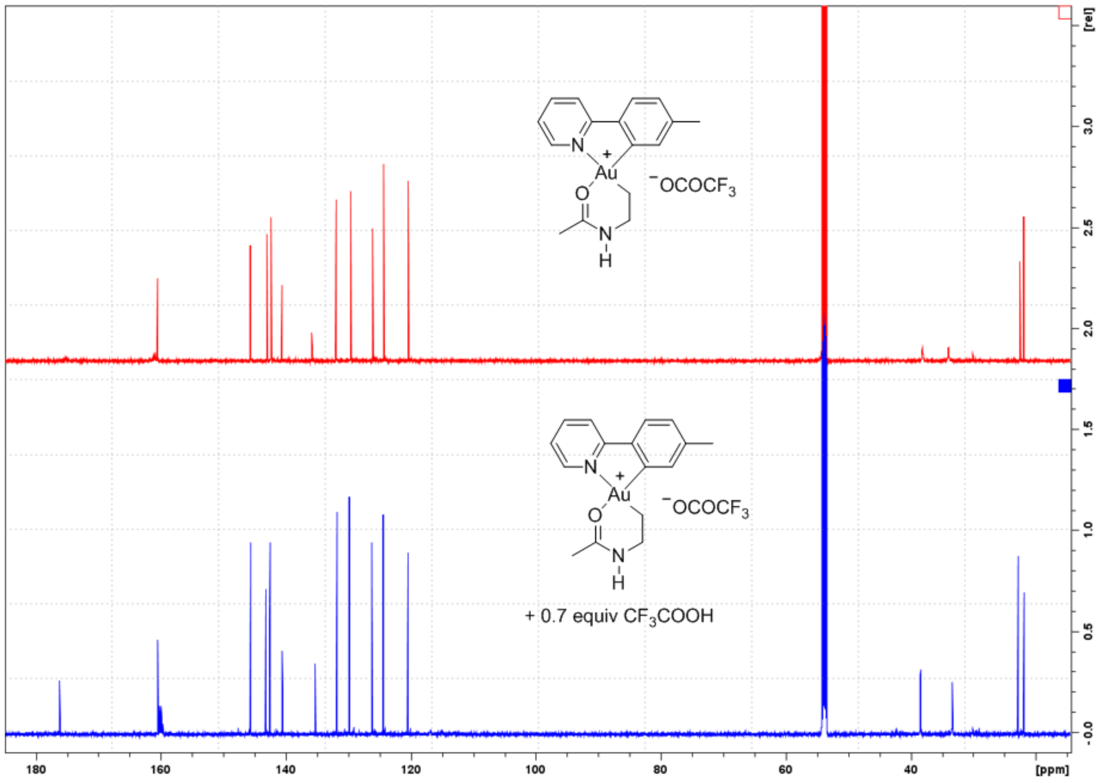

Figure S14. Stacked ^{13}C NMR (600 MHz, CD_2Cl_2) spectra of complex **17**. Top: ^{13}C NMR spectrum of dry **17**. Bottom: ^{13}C NMR spectrum of **17** with *ca.* 0.7 equiv HOAcF added, leading to less broadened resonances.

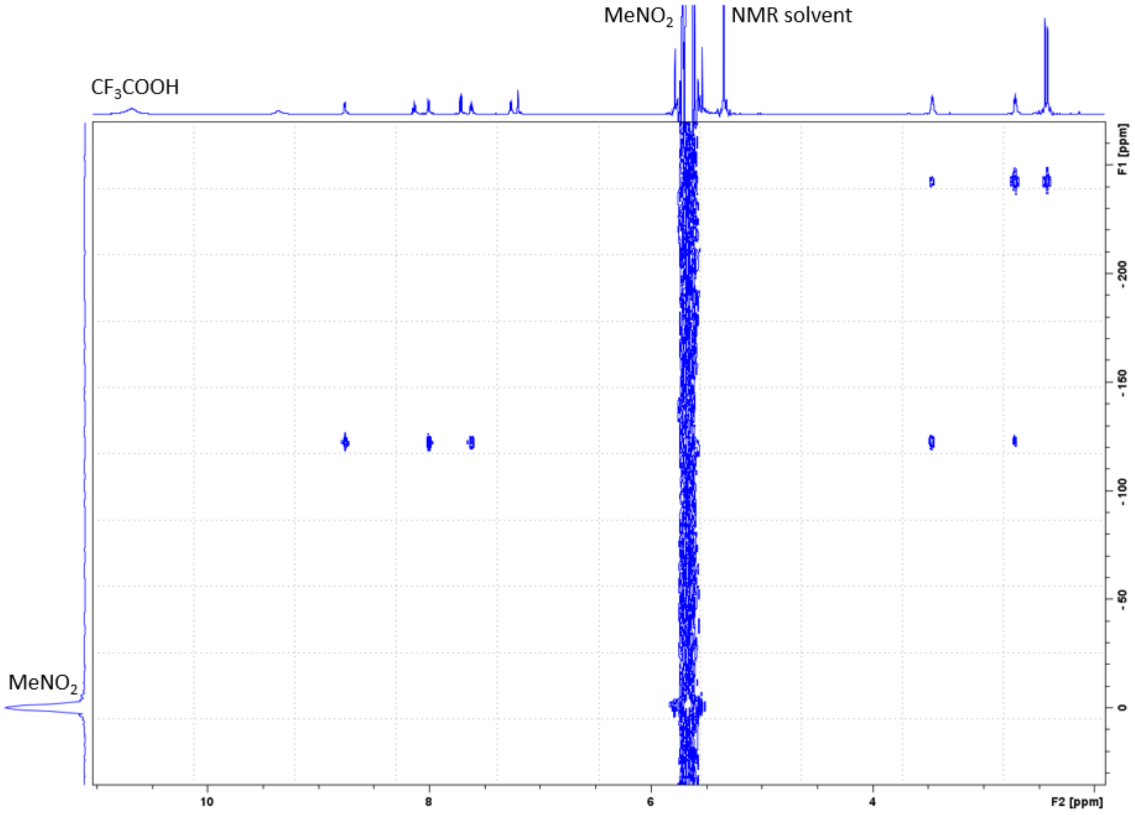

Figure S15. ^{1}H-^{15}N HMBC (600 MHz, CD_2Cl_2) spectrum of complex **17** with *ca.* 0.7 equiv HOAcF added.

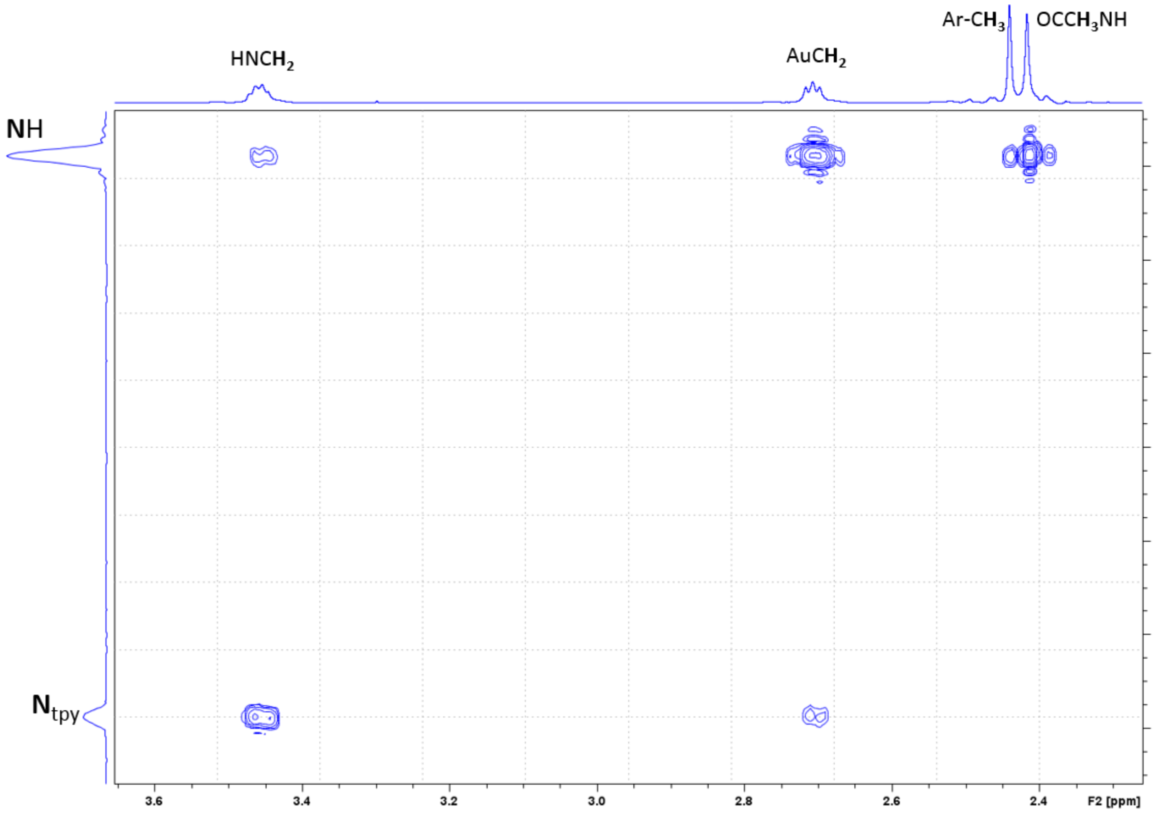

Figure S16. ^{1}H-^{15}N HMBC (600 MHz, CD_2Cl_2) spectrum of complex **17** with *ca.* 0.7 equiv HOAcF added. Close up view of the correlations of the nitrogens with the protons in the aliphatic region.

Computational details

The reaction mechanism proposed for the formation of **16** and **17** is presented in Figure S17. The energies for the intermediates and transition states leading to **16** have been reported[8] but are included in Figure S17 for comparison. The energies for **TS13-A**, **A**, **B**, and **17** were computed with the methodology used in our previous work [PBE0+GD3/6-311+G** for C, H, N, O, F and SDD for Au/SMD(acetonitrile), see xyz supporting file for geometries and free energies in Hartrees].

Based on the calculated energies, the formation of **16** and **17** is explained by the ability of water and acetonitrile to act as nucleophiles in the addition to ethylene (intermediate **A**, Figure S17). In the absence of acid, the addition of water to ethylene is assisted by the OAc^F anion to form intermediate **C**. After this step, which is exergonic by 8.8 kcal mol^{-1}, the coupling of the alcohol and acetonitrile, mediated by Au (*trans* to tpy-*C*) and the OAc^F anion yields the final product **16**. In acidic media, the ability of the OAc^F anion to assist water deprotonation decreases (the OAc^F anion can interact with $HOAc^F$ via H-bonding) allowing the addition of acetonitrile to ethylene, as in a Ritter reaction.[9] Addition of water followed by proton-transfer reactions yields the final product **17**, which is the thermodynamic product (ca 10 kcal mol^{-1} lower in energy than **16**).

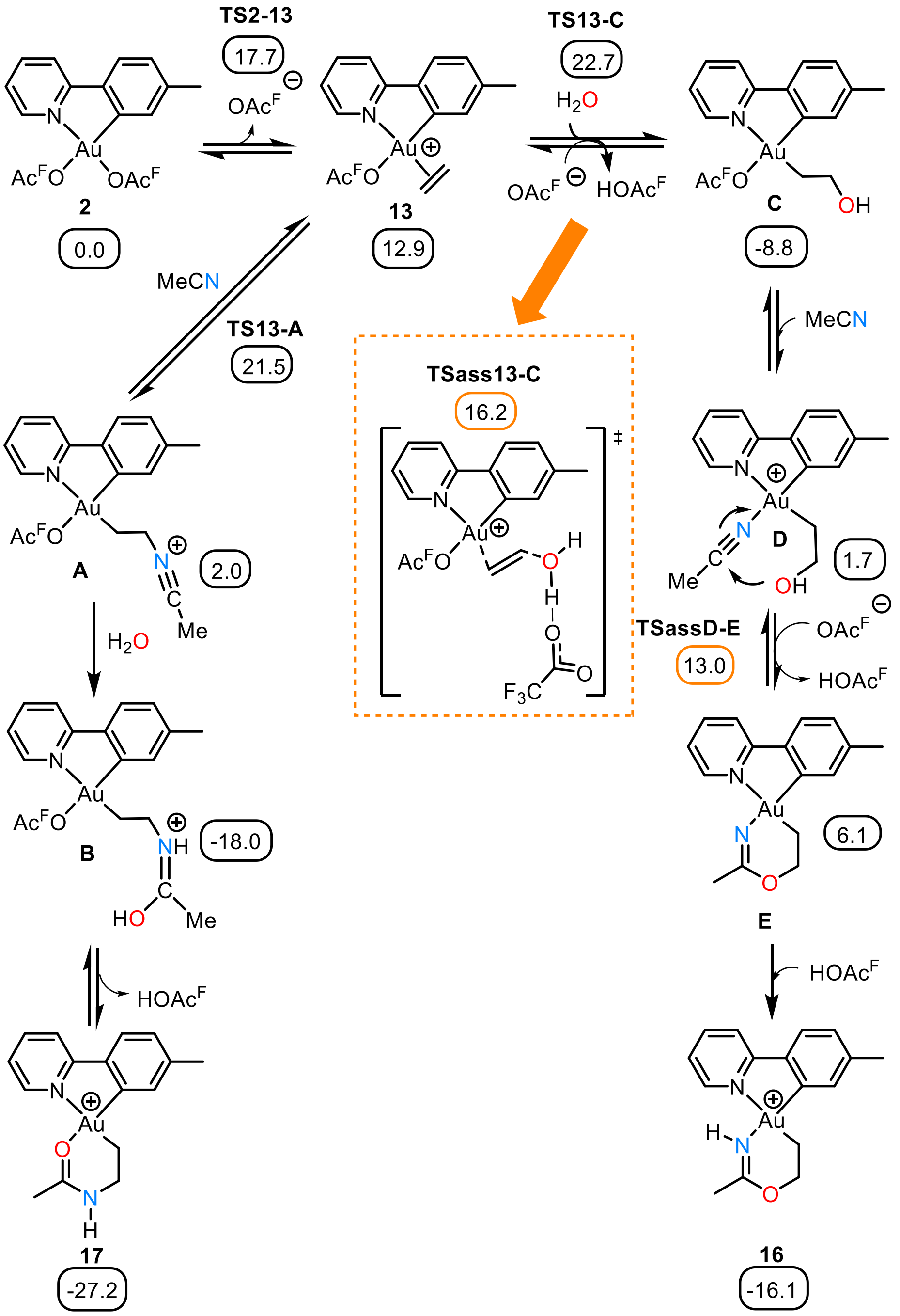

Figure S17. Reaction mechanism proposed for the formation of **16** and **17** ($OAc^F = OCOCF_3$). Free energies obtained from DFT calculations in acetonitrile are given in kcal mol^{-1}.

References

(1) Langseth, E.; Görbitz, C. H.; Heyn, R. H.; Tilset, M. Versatile Methods for Preparation of New Cyclometalated Gold(III) Complexes. *Organometallics* **2012**, *31*, 6567-6571.

(2) *Bruker APEX3, SAINT, SADABS, XPREP Ver. 2016.5-0*, Bruker AXS inc, Madison, Wisconsin, USA, **2016**.

(3) Sheldrick, G. SHELXT - Integrated space-group and crystal-structure determination. *Acta Crystallogr. Sect. A* **2015**, *71*, 3-8.

(4) Sheldrick, G. Crystal structure refinement with SHELXL. *Acta Crystallogr. Sect. C* **2015**, *71*, 3-8

(5) Dolomanov, O. V.; Bourhis, L. J.; Gildea, R. J.; Howard, J. A. K. ; Puschmann, H. *J. Appl. Crystallogr.* **2009**, *42*, 339-341.

(6) Allen, F. H.; Johnson, O.; Shields, G. P.; Smith, B. R.; Towler, M. CIF applications. XV. enCIFer: a program for viewing, editing and visualizing CIFs. *J. Appl. Crystallogr.* **2004**, *37*, 335-338.

(7) Putz, H.; Brandenburg, K. *Diamond - Crystal and Molecular Structure Visualization, Ver. 4.4.0,* Crystal Impact: Kreuzherrenstr. 102, 53227 Bonn, Germany, **1997**.

(8) Holmsen, M. S. M.; Nova, A.; Balcells, D.; Langseth, E.; Øien-Ødegaard, S.; Tråseth, E. A.; Heyn, R. H.; Tilset, M. Small-molecule activation at Au(iii): metallacycle construction from ethylene, water, and acetonitrile. *Dalton Trans.* **2016**, *45*, 14719-14724.

(9) Ritter, J. J.; Minieri, P. P. A New Reaction of Nitriles. I. Amides from Alkenes and Mononitriles. *J. Am. Chem. Soc.* **1948**, *70*, 4045-4048.
